# Dual regulation of cytoplasmic and mitochondrial acetyl-CoA utilization for improved isoprene production in *Saccharomyces cerevisiae*

**DOI:** 10.1038/ncomms12851

**Published:** 2016-09-21

**Authors:** Xiaomei Lv, Fan Wang, Pingping Zhou, Lidan Ye, Wenping Xie, Haoming Xu, Hongwei Yu

**Affiliations:** 1 Institute of Bioengineering, College of Chemical and Biological Engineering, Zhejiang University, Hangzhou 310027, China; 2 Key Laboratory of Biomass Chemical Engineering of Ministry of Education, Zhejiang University, Hangzhou 310027, China

## Abstract

Microbial production of isoprene from renewable feedstock is a promising alternative to traditional petroleum-based processes. Currently, efforts to improve isoprenoid production in *Saccharomyces cerevisiae* mainly focus on cytoplasmic engineering, whereas comprehensive engineering of multiple subcellular compartments is rarely reported. Here, we propose dual metabolic engineering of cytoplasmic and mitochondrial acetyl-CoA utilization to boost isoprene synthesis in *S. cerevisiae*. This strategy increases isoprene production by 2.1-fold and 1.6-fold relative to the recombinant strains with solely mitochondrial or cytoplasmic engineering, respectively. By combining a modified reiterative recombination system for rapid pathway assembly, a two-phase culture process for dynamic metabolic regulation, and aerobic fed-batch fermentation for sufficient supply of acetyl-coA and carbon, we achieve 2527, mg l^−1^ of isoprene, which is the highest ever reported in engineered eukaryotes. We propose this strategy as an efficient approach to enhancing isoprene production in yeast, which might open new possibilities for bioproduction of other value-added chemicals.

## Abstract (web summary)

Microbial production of isoprene from renewable feedstock is a promising alternative to petroleum-based processes. Here, the authors show that isoprene production in the yeast *Saccharomyces cerevisiae* can be improved by dual metabolic engineering of cytoplasmic and mitochondrial acetyl-CoA utilization.

## Graphical abstract

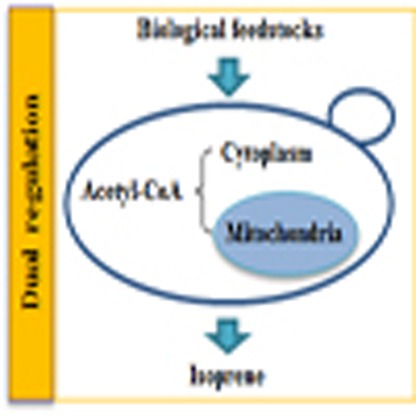

Isoprene, one of the simplest members of the isoprenoids, has wide industrial applications ranging from rubber production to perfume and pesticide manufacturing. Currently, the industrial supply of isoprene is derived entirely from petroleum-derived feedstocks, which is subject to fluctuations in the petroleum market and has also led to serious environmental concerns. Microbial biosynthesis of isoprene provides an attractive and potentially alternative route to the sustainable development of isoprene-related industries. In addition to the common advantages of using microbial systems for production of natural products, such as the ease of pathway engineering and the ability for large-scale fermentation, the use of microbial platforms for isoprene production can also simplify downstream processes due to the volatile and hydrophobic nature of isoprene, which can be easily condensed from the gas phase in bioreactors, with no need for additional purification.

In nature, there are two distinct biochemical pathways involved for production of isoprenoids, the mevalonate (MVA) pathway and methylerythritol phosphate (MEP) pathway^1^,^2^. With some exceptions, the MVA pathway is typically found in eukaryotes, whereas the MEP pathway is present in most bacteria and green algae. Both pathways end with isopentenyl pyrophosphate (IPP) and its isomer dimethyl allyl pyrophosphate (DMAPP), which then undergo condensation, cyclization, oxidation and so on, to generate the diverse carbon skeletons found in terpenoids. Although no *ISPS* gene (encoding isoprene synthase) has been elucidated in any microorganism, overexpression of *ISPS* gene from plants^3^,^4^,^5^ can effectively endow microorganisms with the capability of isoprene production using their own metabolic pathways.

As a well-characterized model organism, *Saccharomyces cerevisiae* is an attractive platform for bioproduction due to its industrial robustness, ease of genetic manipulation and inherent safety. It employs the MVA pathway, consisting of *ERG10* (encoding acetoacetyl-CoA thiolase), *HMGS* (encoding 3-hydroxy-3-methylglutaryl-CoA synthase), *HMG1* (encoding HMG-CoA reductase), *MK/ERG12* (encoding mevalonate kinase), *PMK/ERG8* (encoding phosphomevalonate kinase), *MVD1* (encoding mevalonate pyrophosphate decarboxylase) and *IDI1* (encoding IPP isomerase), to generate essential isoprenoids such as carotenoids, quinones and steroids. As the precursor of the MVA pathway, acetyl-CoA is required for the production of isoprenoids. In yeast, acetyl-CoA is produced and consumed in different compartments, especially the cytoplasm and mitochondria. Recently, much of the work on enhancing isoprenoid production has been targeted to improve acetyl-CoA supply and/or utilization; however, all of these studies focused on cytoplasmic engineering on the account of the MVA pathway being located in cytoplasm, such as overexpression of the rate-limiting enzyme tHMG1 (truncated HMG1)^6^,^7^, downregulation of competing metabolic branches^8^,^9^,^10^, and enhancing acetyl-CoA supply from the cytoplasm^11^,^12^. In contrast, engineering of mitochondrial acetyl-CoA metabolism has largely been ignored. It is generally recognized that under aerobic conditions a great amount of pyruvate is transferred from cytoplasm to mitochondria and converted into acetyl-CoA. Since the membranes of organelles are impermeable to acetyl-CoA, this metabolite cannot be transported directly between different compartments without the carnitine/acetyl-carnitine shuttle^13^. Despite the presence of a carnitine-dependent acetyl-CoA transport system in *S. cerevisiae*, its inability to synthesize carnitine *de nov*o prevents the transport of mitochondrial acetyl-CoA into the cytoplasm^14^. Therefore, improving utilization efficiency of the acetyl-CoA pool in mitochondria has become a key issue for further enhancing isoprenoid production.

In recent years, organelle engineering has attracted increasing attention in the biosynthesis of chemicals. For example, the capacity of tobacco plants for biosynthesis of sesquiterpenes, patchoulol and amopha-4,11-diene was explored by metabolic engineering within the plastid^15^. As much as 100–1,000 times higher production was achieved by plastid engineering as compared with that of traditional engineering reported previously^16^,^17^,^18^. In 2013, Stephanopoulos and co-workers investigated the potential of mitochondrial engineering in isobutanol biosynthesis, achieving higher production of isobutanol by compartmentalization of the Ehrlich pathway into the mitochondria rather than cytoplasm^19^. As an independent subcellular organelle, mitochondrial engineering may provide a potential approach to enhanced production of target isoprenoids for the following reasons: (1) the acetyl-CoA pool in mitochondria could be utilized for isoprenoid production in addition to the cytoplasmic supply; (2) the relatively high reducing redox potential and rich ATP in the mitochondrial matrix might be beneficial for biochemical reactions^20^; (3) the diversion of intermediates into competing pathways could be prevented by the mitochondrial membrane. To take advantage of mitochondrial engineering and make full use of mitochondrial acetyl-CoA in addition to cytoplasmic acetyl-coA, dual metabolic engineering of the MVA pathway in both the mitochondria and the cytoplasm might be a route to enhancing the bioproduction of isoprenoids, including isoprene.

In the present work, we explore the potential capacity of *S. cerevisiae* in isoprene biosynthesis by dual regulation of cytoplasmic and mitochondrial acetyl-CoA utilization. First, the complete isoprene synthetic pathway is assembled in mitochondria by employing a reiterative recombination system featured by rapid marker recycling, and a modified *GAL* regulation system responsive to glucose^21^ is introduced for dynamic regulation of the pathway genes. The effect of mitochondrial engineering on isoprene production is then examined and compared with that of cytoplasmic engineering. The 1.7-fold improvement of isoprene production and 80% reduction of squalene accumulation in mitochondrial engineered strain demonstrate the advantage of mitochondrial engineering over cytoplasmic engineering in reducing the loss of precursor to competing pathway. Finally, mitochondrial engineering and cytoplasmic engineering are combined for comprehensive utilization of acetyl-CoA, and achieve 2.1-fold and 1.6-fold improvement of isoprene production compared with recombinant strains with solely mitochondrial or cytoplasmic engineering, respectively. This strategy offers an efficient approach to enhancing isoprene production in yeast and might also be applicable for bioproduction of other value-added chemicals.

## Results

### Construction of a toolbox for rapid pathway assembly

**Figure 1:**
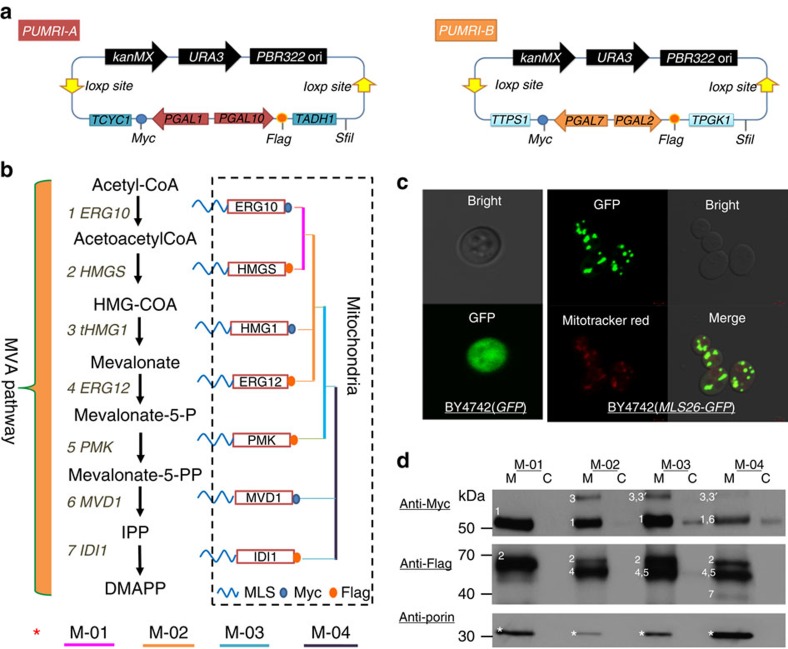
Reconstruction of the MVA biosynthesis pathway in the mitochondria of *Saccharomyces cerevisiae*. (**a**) Plasmids pUMRI-A and pUMRI-B. In each plasmid, two expression cassettes (with different GAL promoters, terminators and C-terminal tags) in reversed orientations were introduced and arranged between the two loxp sites of pUMRI construct. (**b**) Re-localizing the MVA pathway (including seven genes: 1-*ERG10*; 2-*HMGS*; 3-*tHMG1*; 4-*ERG12*; 5-*PMK*; 6-*MVD1*; and 7-*IDI1*) in the mitochondria. Each gene was fused with N-terminal MLS and C-terminal Myc or Flag. (**c**) Fluorescence microscopy of yeast strains expressing GFP (BY4742-PUG35) and MLS-GFP (BY4742-PUG35-MLS26). Scale bar, 2 μm. (**d**) Western blot analysis of recombinant strains: BY4742-M-01 (BY4742 overexpressing ERG10 and HMGS), BY4742-M-02 (BY4742-M-01 overexpressing tHMG1 and ERG12), BY4742-M-03 (BY4742-M-02 overexpressing PMK and tHMG1), BY4742-M-04 (BY4742-M-03 overexpressing MVD1 and IDI1) (Supplementary Fig. 9, full-scanned images). Bands 1–7 correspond to proteins shown in **b**. M represents mitochondrial engineering.

In this work, genomic integration of pathway genes was performed by reiterative recombination. On the basis of the pMRI vectors constructed in our previous study^22^, a modified toolbox ‘pUMRI' (Fig. 1a) was constructed by combining the *KanMX-Cre/loxP* recombination system^22^,^23^ with URA counter-selection to shorten the operation cycle of pathway assembly by simplifying the marker removal process. *KanMX* was adopted as a selection marker in both *Escherichia coli* and *S. cerevisiae*, while *URA3* (encoding orotidine 5-phosphate decarboxylase) was used for converting 5-fluoroorotic acid (5-FOA) to the cytotoxic 5-fluorouracil leading to cell death. Generally, three common steps are involved in each round of recombination, including gene cloning, transformant selection and marker recycling (Supplementary Methods). After gene integration, the cassette of ‘*loxp-kanMX-URA-loxp*' was removed by the low-occurrence event of mitotic recombination between the repeated *loxp-loxp*, and positive colonies could be selected out by marker loss upon URA counter-selection in a one-step process (Supplementary Fig. 1). It is worth mentioning that each recombination cycle could be completed within only 6–7 days. In contrast, 13–14 days are usually required for completion of one recombination using the *Cre/loxp* system. For expression control of genes in *S. cerevisiae*, herein, a series of GAL promoters were tested using the carotene-based strength characterization method proposed in our previous work^21^. Strains with P_*GAL1*_, P_*GAL10*_, P_*GAL2*_ and P_*GAL7*_ produced carotene comparable to that of P_*TEF1*_ or P_*HXT7*_ (two constitutive promoters generally recognized as strong) upon galactose induction, while producing little carotene when glucose was used as the carbon source (Supplementary Fig. 2), thus demonstrating their carbon source-responsive regulation characteristics^23^,^24^. These four GAL promoters were then introduced into the pUMRI cassettes (Supplementary Fig. 3), generating pUMRI-A and pUMRI-B (Fig. 1a), respectively. To determine the efficiency of marker excision using pUMRI, deletion of *DPP1* (encoding diacylglycerol pyrophosphate phosphatase) was performed (Supplementary Methods), which showed high marker removal efficiency (Supplementary Fig. 4) and low recombination frequency between *loxp* repeats (Supplementary Table 1).

### Pathway assembly and mitochondrial compartmentalization

For localizing the genes of the MVA pathway to the mitochondria of *S. cerevisiae*, a 26 AA N-terminal mitochondrial localization signal (MLS) from subunit IV of the yeast cytochrome oxidase (CoxIV) was selected to fuse with the N-terminus of the target genes (Fig. 1b). The first 25 AA were responsible for transporting proteins into the mitochondrial matrix^25^ and Gln26 was used for removal of the signal peptide after positioning, catalysed by a chelator-sensitive matrix-localized protease^26^. Fluorescence microscopy was carried out to verify the subcellular targeting capability of MLS by comparing the fluorescence distribution of yeast strains expressing green fluorescent protein (GFP) without or with MLS (Fig. 1c), using a secondary mitochondria marker ‘Mitotracker red' as reference. The overlapping of the GFP signals and the Mitotracker red signals of BY4742 (MLS26-GFP) demonstrated the correct mitochondrial localization of the MLS-targeted GFP. To avoid illegal recombination of chromosome in the process of multigene integration, decentralized assembly^22^ was conducted by selecting loci from different chromosomes or from the same chromosome but with long distances in between as the homologous arms, such as *LPP1* (ChrIV 1455866-1455042, encoding lipid phosphate phosphatase), *DPP1* (ChrIV 1031419-1030550), *HO* (ChrIV 48031-46271, encoding homothallic switching endonuclease) and *GAL80* (ChrXIII 171594-172901, encoding a transcriptional repressor involved in transcriptional regulation in response to galactose). On the basis of the above design, we localized the entire MVA pathway (including two copies of the limiting gene *tHMG1* and one copy each of other genes) into the mitochondria via one to four successive rounds of recombination (Supplementary Fig. 5A), generating a series of recombinant strains: BY4742-M-01 (BY4742 overexpressing HMGS and ERG10), BY4742-M-02 (BY4742-M-01 overexpressing tHMG1 and ERG12), BY4742-M-03 (BY4742-M-02 overexpressing PMK and tHMG1) and BY4742-M-04 (BY4742-M-03 overexpressing MVD1 and IDI1). The correct pathway assembly of the recombinant strain BY4742-M-04 was verified by PCR amplification of the seven genes (Supplementary Fig. 5B) from randomly picked colonies. In addition, the expression and mitochondrial localization of the pathway enzymes was confirmed via western blot of the mitochondria fractions of the recombinant strains BY4742-M-01/02/03/04, using porin as the internal reference protein in mitochondria (Fig. 1d). Since there was no distinct size difference between MVD1 and ERG10, PMK and ERG12, and the band of IDI1 was hardly visible as shown in the blotting of BY4742-M-04 due to its relatively low expression level when co-expressed with other genes, these three genes (*MVD1, PMK* and *IDI1*) were integrated individually or in pairs in BY4742 using the same plasmid as BY4742-M-04. Our results confirmed their successful expression and mitochondrial localization (Supplementary Fig. 5C).

**Figure 2:**
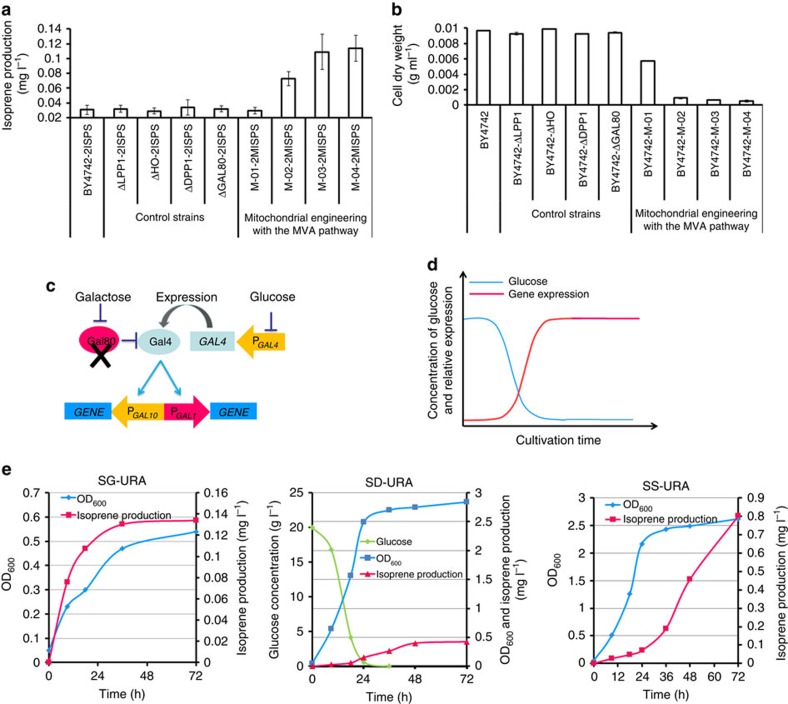
Mitochondrial engineering for isoprene production. (**a**) Production of isoprene in recombinant strains. (**b**) Cell growth of recombinant strains. (**c**) Schematic diagram of a modified *GAL* regulation. (**d**) Two-stage process involved in the growth of GAL80-knockout strains. In the first stage, the genes under control of P_*GAL*_ were expressed at a low level to sustain cell growth; while in the second stage, these genes were overexpressed at a high level. (**e**) Isoprene production and biomass of BY4742-M-04 *MISPS-MISPS* cultured in SG-URA (2% galactose), SD-URA (2% Dextrose) and SS-URA (2% Sucrose). The data in **a**,**b**,**e** are representative of three separate experiments. Bar represents mean±s.d.

The effects of mitochondrial engineering were investigated. First, to examine the possible influence of gene disruption resulting from genomic integration at the corresponding sites, control experiments were conducted by knocking out *LPP1, DPP1, HO* and *GAL80* genes, showing no visible effect on target product accumulation or cell growth. After localization of the isoprene synthetic pathway to the mitochondria, the isoprene production in recombinant strains was significantly improved, especially in BY4742-M-04 *MISPS-MISPS* (Fig. 2a), while the biomass of all BY4742-M-01/02/03/04 strains showed marked decrease (Fig. 2b and Supplementary Fig. 6A). In comparison, the strains overexpressing the corresponding genes in the cytoplasm (BY4742-C-01/02/03/04) mostly showed only slightly slower growth than the control except BY4742-C-04 (Supplementary Fig. 6B), indicating the growth decrease in mitochondria-engineered strains was not only caused by the metabolic burden resulting from multigene overexpression. We hypothesized that the accumulation of cytotoxic metabolic intermediates may have contributed to impaired cell growth. The intermediates of the MVA pathway in BY4742-M-01/02/03/04 could most likely not be transported out of the mitochondria or further converted due to the lack of downstream genes, leading to intermediate accumulation. To verify this hypothesis, pESC-URA-*MISPS-MISPS* was transformed into the mitochondria of BY4742-M-04 (generating BY4742-M-04 *MISPS-MISPS)*, resulting in improved growth on day 4 and thereafter as compared with BY4742-M-04 *MISPS-MISPS* (N498D, inactive enzyme), although its cell growth was still not as good as the control strain (BY4742 *ISPS-ISPS*) (Supplementary Fig. 6C).

To further improve biomass and isoprene production, a two-stage process with varying glucose supply was adopted based on the *GAL* regulation system modified by *GAL80* knock-out^21^ enabling the glucose-responsive control of GAL promoters (Fig. 2c). In the first stage, the genes under control of P_*GAL*_ were expressed at a low level and most of the carbon source was used to sustain cell growth. In the second stage, these genes were overexpressed at a high level to accumulate the fermentation product (Fig. 2d). This way, balanced cell growth and isoprene production were achieved in the *GAL80* deletion strain BY4742-M-04 *MISPS-MISPS*, using glucose or sucrose as the carbon source (Fig. 2e). In the first stage (0–24 h), the strain grew rapidly with little isoprene produced, whereas in the following stage (24–72 h), most of the cellular resources and energy were consumed for isoprene accumulation. Finally, isoprene production in sealed vials was improved to 0.4 and 0.8 mg l^−1^ (with glucose and sucrose, respectively) from 0.13 mg l^−1^ (with galactose), and cell growth was increased by about fivefold. Moreover, since aerobic conditions are essential for mitochondrial acetyl-CoA production and cell metabolism, aerobic batch fermentation was performed with BY4742-M-04 *MISPS-MISPS* using sucrose as the carbon source, which resulted in isoprene production of 108 mg l^−1^ after 3 days.

### Comparison of cytoplasmic and mitochondrial engineering

**Figure 3:**
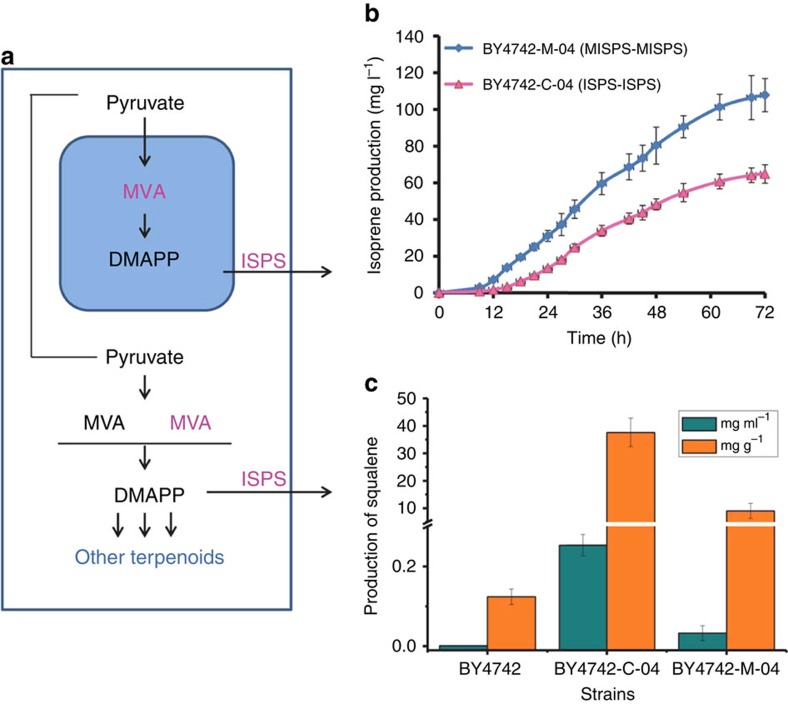
Comparison of cytoplasm-engineered and mitochondria-engineered strains. (**a**) Schematic representation for cytoplasmic engineering and mitochondrial engineering of the complete isoprene synthetic pathway. Gene and pathway reconstruction is marked in magenta. Blue box represents mitochondria. (**b**) Isoprene production of strains constructed by cytoplasmic engineering and mitochondrial engineering of the complete isoprene synthetic pathway in aerobic batch fermentation. (**c**) Squalene production of strains constructed by cytoplasmic engineering and mitochondrial engineering of the MVA pathway. In **b**,**c**, error bars represent s.d. from three independent experiments.

To compare the effect of cytoplasmic engineering and mitochondrial engineering of the MVA pathway (Fig. 3a) on isoprene production, the endogenous MVA pathway was also overexpressed in cytoplasm using pUMRI plasmids, constructing the BY4742-C-01/02/03/04 strains in which all the genes were under control of the same promoters and terminators as BY4742-M-01/02/03/04, with the mitochondrial target sequence as the only difference. The fermentation data demonstrated that the strain overexpressing mitochondria targeted enzymes of the complete MVA pathway together with ISPS, BY4742-M-04 *MISPS-MISPS*, outperformed the corresponding cytoplasm-engineered strain BY4742-C-04 *ISPS-ISPS* in isoprene production (108 mg l^−1^ versus 64 mg l^−1^) (Fig. 3b). To check whether the increased isoprene production in BY4742-M-04 was resulted from the lack of competing downstream pathways in mitochondria, the production of squalene in both strains was analysed. As shown in Fig. 3c, the production of squalene in BY4742-M-04 was lower than that of BY4742-C-04, although the squalene production in both BY4742-M-04 (0.03 mg ml^−1^ and 9.0 mg g^−1^ DCW, Dry Cell Weight) and BY4742-C-04 (0.25 mg ml^−1^ and 37.5 mg g^−1^ DCW) was improved compared with that of the wild-type strain BY4742 (0.001 and 0.12 mg g^−1^ DCW). In addition, expression levels of the pathway enzymes in BY4742-M-04 and BY4742-C-04 were estimated by western blot, with no obvious distinction found in most of the proteins (Supplementary Fig. 7). The above results indicate that the higher isoprene production in mitochondria-engineered strain might be ascribed to the reduction of flux loss into competing pathways by physical separation via the mitochondrial membrane, thus maximizing the flux into the target pathway rather than increasing local enzyme concentrations in the mitochondria.

### Dual regulation of both cytoplasm and mitochondria

**Figure 4:**
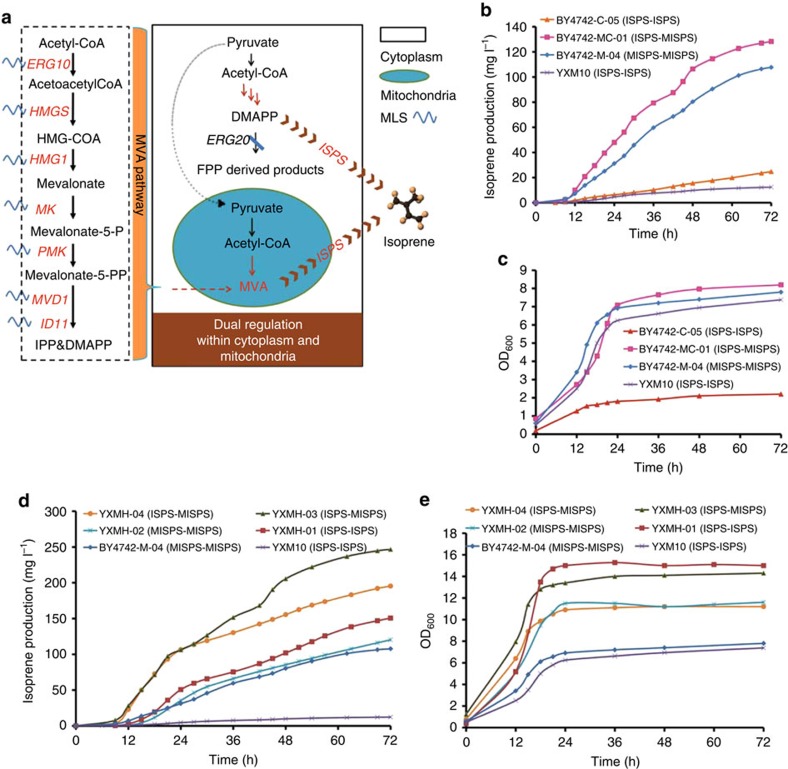
Dual metabolic regulation in the cytoplasm and mitochondria for isoprene production. (**a**) Dual regulation strategy. (**b**) Isoprene production of recombinant strains YXM10 (*ISPS-ISPS*), BY4742-M-04 (*MISPS-MISPS*), BY4742-MC-01 (*ISPS-MISPS*), BY4742-C-05 (*ISPS-ISPS*) in aerobic batch fermentation. (**c**) Growth curves of the recombinant strains (in **b**) in aerobic batch fermentation. (**d**) Isoprene production of recombinant strains YXM10 (*ISPS-ISPS*), BY4742-M-04 (*MISPS-MISPS*), YXMH-01 (*ISPS-ISPS*), YXMH-02 (*MISPS-MISPS*), YXMH-03 (*ISPS-MISPS*), YXMH-04 (*ISPS-MISPS*) in aerobic batch fermentation. (**e**) Growth curves of the recombinant strains (in **d**) in aerobic batch fermentation. YXM10: strain with cytoplasm engineering; BY4742-M-04, strain with mitochondria engineering; BY4742-MC-01, haploid strain with a mixed cytosolic-mitochondrial strategy; BY4742-C-05, haploid strain with comprehensive regulation in the cytoplasm; YXMH-01, the control hybrid strain of YXM10 and BY4742-Δ*Gal80*::*HIS*; YXMH-02, the control hybrid strain of BY4742-M-04-*HIS* and BY4741-Δ*Gal80*::*LEU*; YXMH-03, the hybrid strain of YXM10 and BY4742-M-04-*HIS*; YXMH-04, the hybrid strain of BY4741-C-04-*LEU* and BY4742-M-04-*HIS*.

Since sufficient precursor supply is one of the most essential issues in bio-products accumulation, the complete utilization of acetyl-CoA within multiple compartments might benefit isoprene production in yeast. As previously reported for the cytoplasmic engineering of isoprene-producing *S. cerevisiae*^27^, enhancement of the precursor supply by overexpressing *tHMG1* and downregulation of the competing pathway by weakening the farnesyl pyrophosphate synthetase gene (*ERG20*) led to significant enhancement in isoprene production (generating YXM10), which can be regarded as an effective strategy for cytoplasmic engineering. Therefore, to further improve production of isoprene, dual metabolic regulation of the isoprene synthetic pathway in both cytoplasm and mitochondria was explored (Fig. 4a). One method for dual regulation was to conduct cytoplasmic engineering in addition to mitochondrial engineering in the same strain by overexpressing *tHMG1* and weakening *ERG20* in BY4742-M-04, resulting in BY4742-MC-01. A modest increase in isoprene production (128 mg l^−1^) was obtained in BY4742-MC-01 (*ISPS-MISPS*), compared with strains BY4742-M-04 (*MISPS-MISPS*) (108 mg l^−1^) and YXM10 (*ISPS-ISPS*) (13 mg l^−1^) (Fig. 4b). The same strategy (overexpression of *tHMG1* and downregulation of *ERG20*) was also performed in BY4742-C-04 to generate the cytoplasmic engineering-only control strain BY4742-C-05 (Supplementary Fig. 5D). In contrast to BY4742-MC-01 (*ISPS-MISPS*), BY4742-C-05 (*ISPS-ISPS*) produced as low as 25 mg l^−1^ of isoprene (Fig. 4b) and grew poorly (2.2 OD_600_) under aerobic fermentation condition (Fig. 4c), demonstrating the superiority of dual cytoplasmic-mitochondrial engineering over cytoplasmic engineering alone. However, the dual engineered strain BY4742-MC-01 (*ISPS-MISPS*) was unable to achieve high-density fermentation, especially in the fed phase (data not shown). Therefore, achieving comprehensive regulation of mitochondrial and cytoplasmic acetyl-CoA supply while still preserving high biomass warranted further investigation.

Considering that hybrid strains could generally tolerate higher stresses as compared with haploid strains^28^,^29^,^30^, a hybridization approach was tentatively applied to solve the above-mentioned problem. First, BY4742-M-04-*HIS* was constructed by complementing the *HIS* marker in BY4742-M-04 and then mated with YXM10 to generate YXMH-03. The hybrid strain produced 246 mg l^−1^ of isoprene, which represented approximately 2.3-fold and 19-fold increases in titres relative to the parent strains, respectively (Fig. 4d). Cell growth was also improved by about 180% (Fig. 4e) compared with haploid strains. To further investigate whether the improvement of isoprene production in YXMH-03 was merely caused by cell growth improvement due to the advantages of diploids, two control hybrid strains of YXMH-01 (YXM10 mated with BY4742-Δ*GAL80*) and YXMH-02 (BY4742-M-04 mated with BY4741-Δ*GAL80*) were constructed for comparison. The cell growth of both hybrid strains (YXMH-01, YXMH-02) was improved by ∼1.5–2 folds compared with the corresponding haploid strains (YXM10 and BY4742-M-04) (Fig. 4e). In addition, YXMH-01 (*ISPS-ISPS*) produced 150 mg l^−1^ of isoprene, whereas YXMH-02 (*MISPS-MISPS*) produced 120 mg l^−1^ of isoprene, both higher than that of corresponding parent strains YXM10 (*ISPS-ISPS*) and BY4742-M-04 (*MISPS-MISPS*), but still lower than that of YXMH-03 (*ISPS-MISPS*) (246 mg l^−1^) (Fig. 4d). These results showed that the significant improvement of isoprene production in the strain YXMH-03, 1.6-fold over cytosolic-only engineering (YXMH-01) and 2.1-fold over mitochondrial-only engineering (YXMH-02), was not merely attributed to the better cell growth as a diploid but also the dual regulation of mitochondrial and cytoplasmic acetyl-CoA utilization.

**Figure 5:**
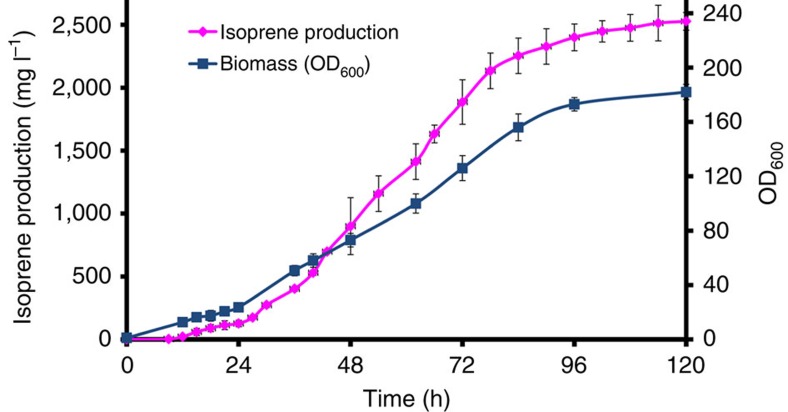
Fed-batch fermentation of YXMH-03 (*ISPS-MISPS*). Fermentation was performed in a 5-l fermentor containing 2.5-l fermentation medium at 30 °C with an airflow rate of 1–3 v.v.m. The pH was controlled automatically at 5.0 with the addition of 5 M NH_4_OH. The isoprene concentration in the off-gas was determined by GC every 3 hours. Error bars represent s.d. from three independent experiments.

Comparison of the two cytoplasmic engineering strains revealed higher isoprene production from BY4742-C-04 than YXM10, suggesting that overexpression of the entire MVA pathway in the cytoplasm could be an alternative strategy for improving isoprene overproduction. To check whether mating of strains respectively overexpressing the entire MVA pathway in the cytoplasm and the mitochondria led to even higher isoprene yield compared with YXMH-03, the entire MVA pathway was reconstructed in BY4741, resulting in BY4741-C-04, complemented with LEU marker, and then mated with BY4742-M-04-*HIS*, generating YXMH-04. Genotype analysis of BY4741-C-04 (Supplementary Fig. 5D) confirmed the successful reconstruction of the MVA pathway in BY4741. YXMH-04 (*ISPS-MISPS*) produced 195 mg l^−1^ of isoprene, which was 80% higher than BY4742-M-04 (*MISPS-MISPS*) but 20% lower than YXMH-03 (*ISPS-MISPS*) (Fig. 4d). In addition, the cell growth of YXMH-04 (*ISPS-MISPS*) (OD_600_=11) was improved by 140% compared with BY4742-M-04 (*MISPS-MISPS*) (OD_600_=7.8), but was still lower than YXMH-03 (*ISPS-MISPS*) (OD_600_=14.3) (Fig. 4e). The lower isoprene production and cell growth of YXMH-04 compared with YXMH-03 might be ascribed to metabolic burden from overexpression of sixteen genes, including four copies of *tHMG1* and two copies of the other six genes involved in the MVA pathway. Finally, fed-batch fermentation was performed with the dual engineered strain YXMH-03 (*ISPS-MISPS*) under aerobic conditions, producing 2527, mg l^−1^ of isoprene within 120 h (Fig. 5).

## Discussion

Biosynthesis of isoprene by microorganisms has drawn considerable attention in the last few years. Current studies are mostly focused on engineering of bacteria, such as *E. coli*, *Bacillus subtilis* and cyanobacteria^4^,^31^,^32^,^33^, through regulation of the native MEP pathway^34^ or introduction of a heterogenous MVA pathway^35^,^36^. In this work, the potential capacity of isoprene biosynthesis in yeast was explored by dual metabolic engineering of the MVA pathway in cytoplasm and mitochondria. Isoprene production of 2527, mg l^−1^ was obtained, which is (to the best of our knowledge) the highest ever reported in engineered eukaryotes.

To introduce the complete MVA pathway into the mitochondria of *S. cerevisiae*, reiterative recombination was utilized, facilitated by a set of marker recyclable integrative tools (pUMRI series) designed for *S. cerevisiae*. In this design, a modified marker recycling system was integrated using a decentralized assembly strategy to reduce labour required for the traditional *Cre/loxP* system and to avoid unwanted deletion from single-locus assembly. In addition, four GAL promoters were chosen based on their high strength and adopted for construction of the pURMI toolbox. Among the synthetic regulatory networks developed in yeast^37^,^38^,^39^, the GAL regulation network is often chosen as a preferred regulator owing to its easy operation. Due to economic concerns, the GAL regulation network has been modified into a glucose-regulating system by *GAL80* deletion and successfully applied for high-yield production of various natural products^22^,^40^,^41^. The production improvement of isoprene and relief of growth inhibition demonstrated the potential of pUMRI as a common ready-to-use toolbox for rapid pathway assembly as well as dynamic metabolic engineering. In spite of these advantages, endeavours are still needed to further upgrade this toolbox. Although the combination of decentralized assembly and mitotic recombination have largely reduced the frequency of unwanted recombination, a low frequency of chromosome rearrangement events still occurred due to repeated *loxp* segments. To circumvent this problem, seamless recombination methods^42^ may be developed and employed in future.

We proposed a metabolic dual engineering strategy based on the pUMRI toolbox targeted at the MVA pathway in both the cytoplasm and mitochondria to make full use of the precursor acetyl-coA and thus enhance isoprene production. Metabolic engineering of cytoplasmic pathways to create industrial yeast strains for isoprenoid production has been frequently explored^43^,^44^, however no report about the engineering of acetyl-CoA utilization in the mitochondria to enhance isoprenoid synthesis has been released. Considering the rich resource of acetyl-coA in mitochondria that cannot be utilized by cytoplasmic enzymes, it was hypothesized that the introduction of downstream genes into mitochondria would utilize this precursor and ultimately increase the production of the target product. Compartmentalization of the MVA pathway together with the isoprene biosynthetic branch into the mitochondria resulted in enhanced isoprene production, demonstrating the potential capacity of mitochondrial engineering for isoprenoid biosynthesis. Interestingly, targeting part of the MVA pathway into mitochondria also resulted in distinct isoprene improvement, though there is no direct evidence for the mitochondrial localization of any MVA pathway enzymes in wild-type yeast^45^,^46^,^47^. In a previous subcellular compartmentation study on the production of plant terpenoids in yeast^48^, increased levels of sesquiterpenes were also observed when the enzymes downstream, of DMAPP and IPP (farnesyl diphosphate synthase, valencene synthase and amorpha-4,11-diene synthase) were introduced into the mitochondria, indicating the presence of a significant pool of DMAPP and IPP in the mitochondria. Moreover, the occurrence of crosstalk between cytosolic and plastidial pathways of isoprenoid biosynthesis has been proposed in plants like *Arabidopsis thaliana*^49^. Improved squalene production was observed from the mitochondria-engineered strain in our study, suggesting that trafficking of isoprenoid intermediates between the organelles and the cytosol is possible, and could be mediated by specific, undiscovered metabolite transporter(s).

In spite of the enhanced isoprene production, compartmentalization of the MVA pathway into the mitochondria led to serious decrease of cell growth, especially in BY4742-M-02/03/04. Upon overexpression of the downstream gene *ISPS*, growth inhibition was slightly relieved, indicating possible occurrence of cytotoxicity ascribed to the accumulation of intermediates in the absence of downstream enzymes. In a previous study on engineering *E. coli* for production of terpenoids through a heterogeneous MVA pathway^50^, precursor-dependent cytotoxicity was reported when the carbon flux through the exogenous pathway was increased by adding extra mevalonate, and the recombinant strains resumed normal growth when the downstream flux was strengthened. Evidence of isoprenoid precursor toxicity has also been reported in *Bacillus subtilis*^51^. To relieve the cytotoxicity caused by MVA pathway reconstruction, we tried to strengthen the downstream isoprene flux by improving the catalytic activity of ISPS through rational design based on sequence alignment with similar terpene synthases, but unfortunately no activity improvement was obtained in the mutant strains (Supplementary Methods and Supplementary Fig. 8). Subsequently, a two-stage process with varying glucose supply was adopted based on a modified *GAL* regulation system^21^ to balance isoprene production and the cell growth. As expected, both the biomass and the isoprene production were improved.

Moreover, when mitochondrial engineering and cytoplasmic engineering of the isoprene synthetic pathway were compared, higher isoprene production was detected in mitochondria-engineered strains. Judging from the results of western blot, the expression levels of most of the enzymes in the mitochondria and in the cytoplasm were not significantly distinct. Therefore, it seems productivity improvements in the mitochondria-engineered strain were not the result of better enzyme expression in the mitochondria. To further explore the reason behind the enhanced isoprene production after mitochondrial localization, isoprene and squalene production were compared between the mitochondria-engineered and cytoplasm-engineered strains. The higher isoprene production and lower squalene production detected in the mitochondria-engineered strains indicated that the mitochondrial compartmentation of the pathway reduced the flux to the competing pathway by keeping competitive enzymes away from the enhanced precursor.

Finally, to maximize the utilization of precursor supply for isoprene production, cytoplasmic engineering was performed in addition to mitochondrial compartmentalization. In dual regulation within a haploid strain, the higher production of isoprene in BY4742-MC-01 (*ISPS-MISPS*) compared with BY4742-M-04 (*MISPS-MISPS*) and YXM10 (*ISPS-ISPS*) suggested that the combination of cytoplasmic and mitochondrial engineering could improve precursor utilization for isoprene production. The lower production of isoprene and worse growth of BY4742-C-05 compared with BY4742-MC-01 indicated that a mixed cytosolic-mitochondrial strategy was superior to cytoplasmic engineering alone. It was speculated that restraining the downstream competing pathway in addition to the overexpression of the complete MVA pathway in the cytoplasm led to accumulation of toxic intermediates (such as IPP/DMAPP), while the mixed cytosolic-mitochondrial strategy with the complete MVA pathway overexpressed in the mitochondria and ΔP_*ERG20*_-*ERG20*::P_*HXT1*_-*ERG20*-P_*TEF1*_-*tHMG1* in the cytoplasm resulted in better cell health, possibly due to the separation of IPP/DMAPP pools by the mitochondrial membrane. In addition, all hybrid strains showed higher biomass compared with that of haploid strains, demonstrating the potential advantage of diploid hybridization in high-density fermentation. Based on previous studies, the cell mass improvement of the diploid strains might be due to: (i) more resource is used for cellular reproduction in diploid strains as indicated by the significantly reduced cell wall components in diploid cells compared with haploid yeast^52^; or (ii) diploid strains can generally tolerate higher stresses as compared with haploid strains, with higher transcript levels in genes responsive to ethanol stress, electron transport, vesicle formation, heavy metal ion transport and metabolism^28^,^29^,^30^. Furthermore, changes in isoprene production between YXMH-01 and YXM10, as well as YXMH-02 and BY4742-M-04, suggested that hybridization may affect the metabolism of the parent strains to some extent. For instance, the control hybrid strain YXMH-01 (*ISPS-ISPS*) showed about 11-fold improvement over the parent strain YXM-10 (*ISPS-ISPS*), which may be ascribed to a combination of improved cell health as well as optimization of the restrained downstream competing pathway due to hybridization. Although downregulation of *ERG20* has been proved as an efficient strategy for improving production of isoprene, the excessive decrease of farnesyl pyrophosphate upon replacement of P_*ERG20*_ with P_*HXT1*_ also repressed the synthesis of sterols, dolichols and geranylgeranyl diphosphate, which are necessary for membrane construction, cell wall synthesis and protein prenylation^53^,^54^. The synthesis repression of these important isoprenoids could adversely influence cell metabolism as well as isoprene production. By hybridizing YXM10 with the wild-type BY4742, the strength of downregulation might be adjusted to a more optimal state so as to improve isoprene production. In summation, it was found that dual regulation of cytoplasmic and mitochondrial acetyl-CoA utilization greatly benefits isoprene production and that mating of haploid strains with distinct pathway manipulation can be a promising strategy for improving cell growth and fermentation productivity. Specially, the advantages of a dual engineered diploid strain include: (i) the targeting of the MVA pathway into mitochondria makes utilization of the rich acetyl-CoA pool in the mitochondria possible, and ensures sufficient precursor supply together with cytoplasmic engineering; (ii) the mitochondrial membrane acts to separate the target pathway from the competing pathways in cytoplasm, thus reducing the diversion of the metabolic intermediates into competing pathways and maximizing the flux directed into the target pathway; (iii) the mitochondrial membrane splits the IPP/DMAPP pools in two, which might help relieve cellular burden due to accumulation of excessive cytotoxic intermediates; and (iv) dual engineered diploid strain generated by mating of separately engineered haploids has higher stress tolerance and improved cell health over haploid strains. However, even though strategies such as mating and dynamic regulation of the pathway genes effectively relieved cell growth inhibition caused by the accumulation of intermediates, growth defects from cytotoxicity were still observed during fermentation. Directed evolution of ISPS using a high-throughput screening method developed based on DMAPP toxicity relief might provide a potential solution to address this problem.

In conclusion, dual metabolic regulation of mitochondria and cytoplasm showed effectiveness in improving isoprene biosynthesis by *S. cerevisiae.* Simultaneous metabolic engineering of different intracellular compartments might open new possibilities for the bioproduction of valuable natural compounds.

## Methods

### Strains, media and reagents

*S. cerevisiae* strain BY4742 (*MATα his3*Δ*1 leu2*Δ*0 lys2*Δ*0 ura3*Δ*0*)^55^ was used as the host for DNA integration and biosynthetic pathway engineering. *E. coli* DH5α (Novagen, USA) was used for gene cloning. LB (Luria-Bertani broth) medium with antibiotics (100 μg ml^−1^ of ampicillin or 50 μg ml^−1^ of kanamycin) was used for cultivation of recombinant *E. coli*. YPD medium (1% yeast extract, 2% peptone and 2% glucose) and YPG medium (1% yeast extract, 2% peptone and 2% D-galactose) were used for cultivation of yeast. SD-URA (synthetic complete drop-out medium with 2% D-glucose and without uracil), SS-URA (synthetic complete drop-out medium with 2% D-sucrose and without uracil) and SG-URA (synthetic complete drop-out medium with 2% galactose and without uracil) were used for selection and cultivation of recombinant strains harbouring the pESC-URA (Stratagene)-derived plasmids. YPD medium containing 200 μg ml^−1^ geneticin (G418) was used for selection of yeast strains with *KanMX* marker. SD-FOA (SD medium with 0.1% w v^−1^ 5-fluoroorotic acid) was used for selection of yeast strains with *KanMX-URA-PRB322ori* marker excision. All restriction enzymes, T4 DNA ligase, DNA polymerase and primers were purchased from Sangon Biotech (Shanghai, China). The standard isoprene, antibiotics and chemicals were purchased from Sigma (Sigma Aldrich, USA).

### Plasmid construction

For construction of the pUMRI toolbox, a series of strong promoters were investigated using the promoter strength reporting system established in our previous work^21^. The construction procedure of P416XWP-13/14/15-*CrtE* was provided in Supplementary Fig. 2A. These recombinant plasmids were transformed into a β-carotene production strain YXWP41 (ref. 21) to compare the promoter strength using carotenoids as a colorimetric reporter. The pUMRI toolbox was modified from the pMRI backbone plasmid (like pMRI-21/35) constructed previously^22^, in which all necessary backbone components were amplified from PUG6 (ref. 56), p416GPD (ref. 57), and pESC-URA (Stratagene), respectively. The *URA* expression cassette was excised from pESC-URA and integrated between the *PBR322ori* and *KanMX* elements in pMRI, generating pUMRI. Specially, pUMRI-A was modified based on pMRI-21 and pUMRI-B was modified based on pMRI-35, in which *GAL2* and *GAL7* promoters were introduced. The detailed construction procedure and primers are provided in supporting information (Supplementary Fig. 3 and Supplementary Table 2). For characterization of the marker excision strategy involved in this protocol, pUMRI-A-*DPP1* was constructed, in which *DPP1* homologous arms were amplified with primers of *DPP*-UPF1, *DPP*-UPR1, *DPP*-DF1, *DPP*-DR1 and inserted into the pUMRI-A. Genotype identification of recombinant strains (BY4742-01^+^ and BY4742-01) was performed with the primers *DPP1*-UPF2 and *GAL1*F (Supplementary Table 2). Besides, the 26 AA N-terminal mitochondrial localization signal (MLS) from subunit IV of the yeast cytochrome oxidase (CoxIV) (GenBank No. 001181052) was amplified from the genome of BY4742 and inserted into PUM35 (Youbio, China) to generate PUM35-*MLS*. The same signal sequence was introduced into the previously constructed pESC-URA*-ISPS-ISPS* (ref. 27) to generate pESC-URA*-ISPS-MISPS* (one copy of MLS) and pESC-URA*-MISPS-MISPS* (two copy of MLS).

### Strain construction

The main *S. cerevisiae* strains used in this study are listed in Table 1. The complete list of strains and plasmids are presented in Supplementary Table 3. Primers used for strain construction are listed in Supplementary Table 2. As the control, the *LPP1/DPP1/HO/GAL80*-knockout stains were obtained by cloning the corresponding genes into pUMRI plasmids and integrating into BY4742. BY4742-M-01/02/03/04 were constructed by integrating the genes of the entire MVA pathway including *ERG10*, *HMGS*, *HMG1*, *MK/ERG12*, *PMK/ERG8*, *MVD1* and *IDI1* into the genome of BY4742 using pUMRI-*MLS* plasmids (Supplementary Methods). The same process was also performed for construction of BY4742-C-01/02/03/04 and BY4741-C-04 using pUMRI plasmids. The dual regulation strain of BY4742-MC-01 or BY4742-C-05 was obtained by cloning *tHMG1* gene into the pMRI-28-P_ERG20_ plasmid constructed in our previous study^27^, followed by integration into BY4742-M-04 or BY4742-C-04. BY4742-M-04-*HIS* was constructed by complementation of the auxotroph marker *HIS* in BY4742-M-04. The hybrid strain YXMH-01 was obtained by mating BY4742-Δ*GAL80*::*HIS* (LYS^−^, LEU^−^, URA^−^), YXM10 (MET^−^, HIS^−^, URA^−^) and selecting on SD-LYS-LEU-MET-HIS (synthetic defined medium with glucose as carbon source and lysine, leucine, methionine and histidine omitted). The hybrid strain YXMH-02 was obtained by mating BY4742-M-04-*HIS* (LYS^−^, LEU^−^, URA^−^) and BY4741-Δ*GAL80::LEU* (MET^−^, HIS^−^, URA^−^). The hybrid strain YXMH-03 was obtained by mating BY4742-M-04-*HIS* (LYS^−^, LEU^−^, URA^−^) and YXM10 (MET^−^, HIS^−^, URA^−^). The hybrid strain YXMH-04 was obtained by mating BY4741-C-04-*LEU* and BY4742-M-04-*HIS*. For production of isoprene, the pESC-URA vectors harbouring *ISPS* were transformed in the recombinant strains.

### Localization of MLS-GFP by fluorescence microscopy

To test the mitochondrial targeting capabilities of MLS from subunit IV of the yeast cytochrome oxidase, yeast cells expressing GFP and MLS-GFP were observed by fluorescence microscopy. Before imaging, cells of BY4742 (PUG35-*MLS*) were stained with 1 μM Mitotracker red (Invitrogen, USA) for 40 min and washed in PBS. Light and epifluorescence microscopic examinations were performed, respectively, using an Eclipse 80i microscope (Nikon) and a ZEISS LSM780 inverted confocal microscope (Carl Zeiss Inc.).

### Western blot analysis

To check the expression or positioning of tagged proteins, yeast cells were cultured in YPG medium, collected at OD 10.0 and analysed by western blot according to the method described by Sui *et al*.^58^. The cytoplasmic and mitochondrial fractions of engineered strains were separated using a mitochondria extraction kit (Bioversion). Total protein in full cells were extracted using a yeast protein extraction kit (Sangon Biotech, China). For comparison of the protein expression in cytoplasm and mitochondria engineering strains, total protein in full cells and each subcellular fraction were measured using a BCA protein assay kit (Sangon Biotech, China) and 4 μg of protein was loaded in each lane. The protein samples were fractionated on 12% SDS–polyacrylamide gel electrophoresis gel and transferred to polyvinylidene difluoride membranes (Millipore). The membranes were incubated with the indicated primary and secondary antibodies (anti-myc, Clontech; anti-porin, Invitrogen; anti-flag, MBL). Proteins were ultimately visualized by enhanced chemiluminescence and autoradiography (ECL; Thermo Scientific, Waltham, MA, UK).

### Quantification of isoprene and squalene

For analysis of the assembled isoprene pathway, the BY4742-derived strains harbouring pESC-URA-*ISPS/MISPS* were cultivated in 3 ml SD-URA medium at 30 °C with shaking (200 r.p.m.). Then the overnight-grown seed culture was inoculated into sealed vials (17 ml) containing 5 ml fresh SG/SS/SD-URA medium to an initial OD_600_ of 0.05 and incubated under the same conditions for three days. Then the gas sample was collected from the headspace of the sealed culture with a gas-tight syringe, and analysed by a GC system equipped with an HP-FFAP column (30 m × 0.25 mm, 0.25 μm film thickness) and a flame ionization detector (FID). The temperature of oven, injector and detector was held at 80 °C, 180 °C and 180 °C, respectively. The peak area was converted to isoprene concentration using a calibration curve, which was prepared by adding various amounts of isoprene standard (Aladdin, Shanghai, China) into 5 ml SG-URA medium under the same conditions for cultivation.

For quantification of squalene, strains of BY4742, BY4742-C-04, BY4742-M-04 were cultivated in 5 ml YPD medium at 30 °C with shaking (200 r.p.m.). Then ∼2% of the overnight-grown seed culture was inoculated into 50 ml fresh YPG medium to an initial OD_600_ of 0.05 and incubated under the same conditions for another 120 h. Cells were harvested from 2 ml of culture by centrifugation at 4,000*g*, washed twice, and then dried at 95 °C to a constant weight for measuring the dry cell weight. Another 2 ml of culture was harvested by centrifugation and lysed by adding 700 μl of 3 M hydrochloric acid and heating at 100 °C for 3 min. After centrifugation, the cell pellet was washed twice with distilled water and extracted with acetone by vortexing for 15 min (ref. 27). The analysis of squalene was performed by HPLC (Agilent 20AT) equipped with a Kromasil C18 column (4.6 mm × 250 mm) and the signal was detected by a UV detector at 195 nm. The mobile phase was 100% acetonitrile with a flow rate of 1 ml min^−1^ at 40 °C.

### Fed-batch fermentations

The media used for fed-batch fermentation were based on media described by van Hoek *et al*.^59^, composed of 25 g l^−1^ sucrose, 15 g l^−1^ (NH4)_2_SO_4_, 8 g l^−1^ KH_2_PO4, 3 g l^−1^ MgSO_4_, 0.72 g l^−1^ ZnSO_4_·7H_2_O, 12 ml vitamin solution and 10 ml trace metal solution. Seed cultures for bioreactors were prepared by inoculating 100 μl of frozen cells in 20% (vol vol^−1^) glycerol into 20 ml vials containing 5 ml of SD-URA medium. Seed cultures were subcultured after ∼24 h of growth at 30 °C by transferring 1 ml of culture into 500 ml flasks containing 100 ml fermentation media. Three seed flasks were grown for about 24 h to an OD_600_ of 4–8 and then transferred to a 5 l fermentor (Shanghai Huihetang Bioengineering Equipment CO. Ltd, China) containing 2.5 l fermentation media. Fermentation was carried out at 30 °C with an agitation speed of 400 r.p.m. and an airflow rate of 1–3 v.v.m. The pH was maintained at pH 5.0 by automatic addition of 5 M NH_4_OH. The isoprene concentration in the off-gas was determined by GC every 3 h and measured in triplicate.

### Data availability

The data that support the findings of this study are available from the corresponding author on request.

## Additional information

**How to cite this article**: Lv, X. *et al*. Dual regulation of cytoplasmic and mitochondrial acetyl-CoA utilization for improved isoprene production in *Saccharomyces cerevisiae*. *Nat. Commun.* 7:12851 doi: 10.1038/ncomms12851 (2016).

## Supplementary Material

Supplementary Information Supplementary Figures 1-9, Supplementary Tables 1-3, Supplementary Methods and Supplementary References.

## Figures and Tables

**Table 1:** List of the main strains in this study*.

**Strains**	**Genotype**	**Source**
BY4742	*MATα, his3*Δ*1, leu2*Δ*0, lys2*Δ*0, ura3*Δ*0*	Ref. 55
BY4742-01+	BY4742, Δ*DPP1*::T_*ADH1*_-P_*GAL10*_-P_*GAL1*_-T_*CYC1*_-(*Kan*MX-*URA3-PBR322ori*)	This study
BY4742-01	BY4742, ΔDPP1::T_*ADH1*_-P_*GAL10*_-P_*GAL1*_-T_*CYC1*_	This study
BY4742-C-01	BY4742, Δ*LPP1:* T_*CYC1*_*-ERG10*-P_*GAL1*_-P_*GAL10*_*-HMGS*-T_*ADH1*_	This study
BY4742-C-02	BY4742-C-01, Δ*HO:* T_*TPS1*_*-tHMG1*-P_*GAL7*_-P_*GAL2*_*-ERG12*-T_*PGK1*_	This study
BY4742-C-03	BY4742-C-02, Δ*DPP1:* T_*CYC1*_*-tHMG1*-P_*GAL1*_-P_*GAL10*_*-PMK*-T_*ADH1*_	This study
BY4742-C-04	BY4742-C-03, Δ*GAL80:* T_*TPS1*_*-MVD1*-P_*GAL7*_-P_*GAL2*_*-IDI1*-T_*PGK1*_	This study
BY4741-C-04	BY4741, Δ*LPP1:* T_*CYC1*_*-ERG10*-P_*GAL1*_-P_*GAL10*_*-HMGS*-T_*ADH1*_; Δ*HO:* T_*TPS1*_*-tHMG1*-P_*GAL7*_-P_*GAL2*_*-ERG12*-T_*PGK1*_; Δ*DPP1:* T_*CYC1*_*-tHMG1*-P_*GAL1*_-P_*GAL10*_*-PMK*-T_*ADH1*_; Δ*GAL80:* T_*TPS1*_*-MVD1*-P_*GAL7*_-P_*GAL2*_*-IDI1*-T_*PGK1*_	This study
BY4742-M-01	BY4742, Δ*LPP1:* T_*CYC1*_*- ERG10-MLS*-P_*GAL1*_-P_*GAL10*_*-MLS-HMGS*-T_*ADH1*_	This study
BY4742-M-02	BY4742-M-01, Δ*HO:* T_*TPS1*_*- tHMG1-MLS*-P_*GAL7*_-P_*GAL2*_*-MLS-ERG12*-T_*PGK1*_	This study
BY4742-M-03	BY4742-M-02, Δ*DPP1:* T_*CYC1*_*- tHMG1-MLS*-P_*GAL1*_-P_*GAL10*_*-MLS-PMK*-T_*ADH1*_	This study
BY4742-M-04	BY4742-M-03, Δ*GAL80:* T_*TPS1*_*- MVD1-MLS*-P_*GAL7*_-P_*GAL2*_*-MLS-IDI1*-T_*PGK1*_	This study
BY4742-MC-01	BY4742-M-04, ΔP_*ERG20*_*-ERG20*::P_*HXT1*_*-ERG20*-P_*TEF1*_*-tHMG1*	This study
BY4742-C-05	BY4742-C-04, ΔP_*ERG20*_*-ERG20*::P_*HXT1*_*-ERG20*-P_*TEF1*_*-tHMG1*	This study
YXM10	BY4741, (*HMG1*)::*tHMG1*, ΔP_*ERG20*_::P_*HXT1*_, Ty4::*ERG10-ACS2*, Δ*GAL80::LEU2*	Ref. 27
YXMH-01	Diploid BY4742-Δ*GAL80::HIS* xYXM10	This study
YXMH-02	Diploid BY4742-M-04-*HIS* x BY4741-Δ*GAL80::LEU*	This study
YXMH-03	Diploid BY4742-M-04*-HIS* x YXM10	This study
YXMH-04	Diploid BY4742-M-04-*HIS* x BY4741-C-04-LEU	This study

^*^The complete list of strains and plasmids are presented in Supplementary Table 3.
