# Supplementary material for: Dual regulation of cytoplasmic and mitochondrial acetyl-CoA utilization for improved isoprene production in Saccharomyces cerevisiae
Source: Nat Commun. 2016 Sep 21;7:12851. doi: 10.1038/ncomms12851 (PMC5036000; doi:10.1038/ncomms12851)
Supplement: Supplementary Information — Supplementary Figures 1-9, Supplementary Tables 1-3, Supplementary Methods and Supplementary References. [file ncomms12851-s1.pdf]

SUPPLEMENTARY INFORMATION

Supplementary Figures

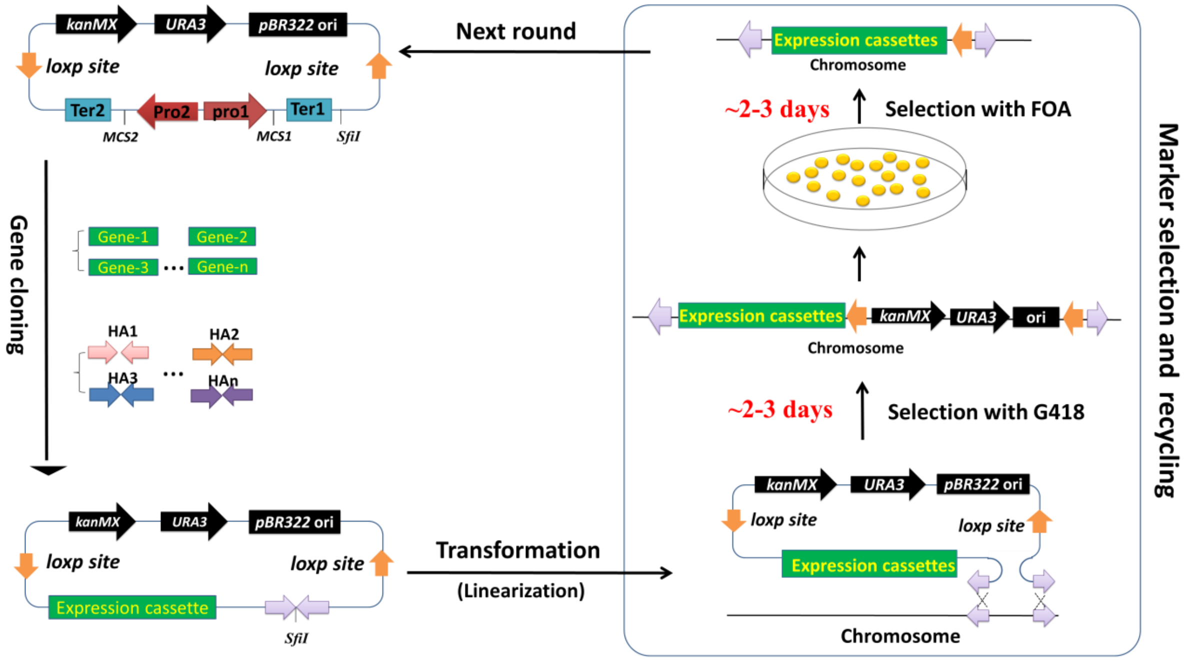

Supplementary Figure 1. Schematic of marker recycling in reiterative recombination.

Each round of recombination consisted of two steps, including genes assembly and recycling. Double selection (G418 and FOA) was adopted for rapid marker recycling. HA indicates homologous arms. For decentralized assembly, HAs were selected from sites on different chromosomes or the same chromosome but with a long distance in between.

A

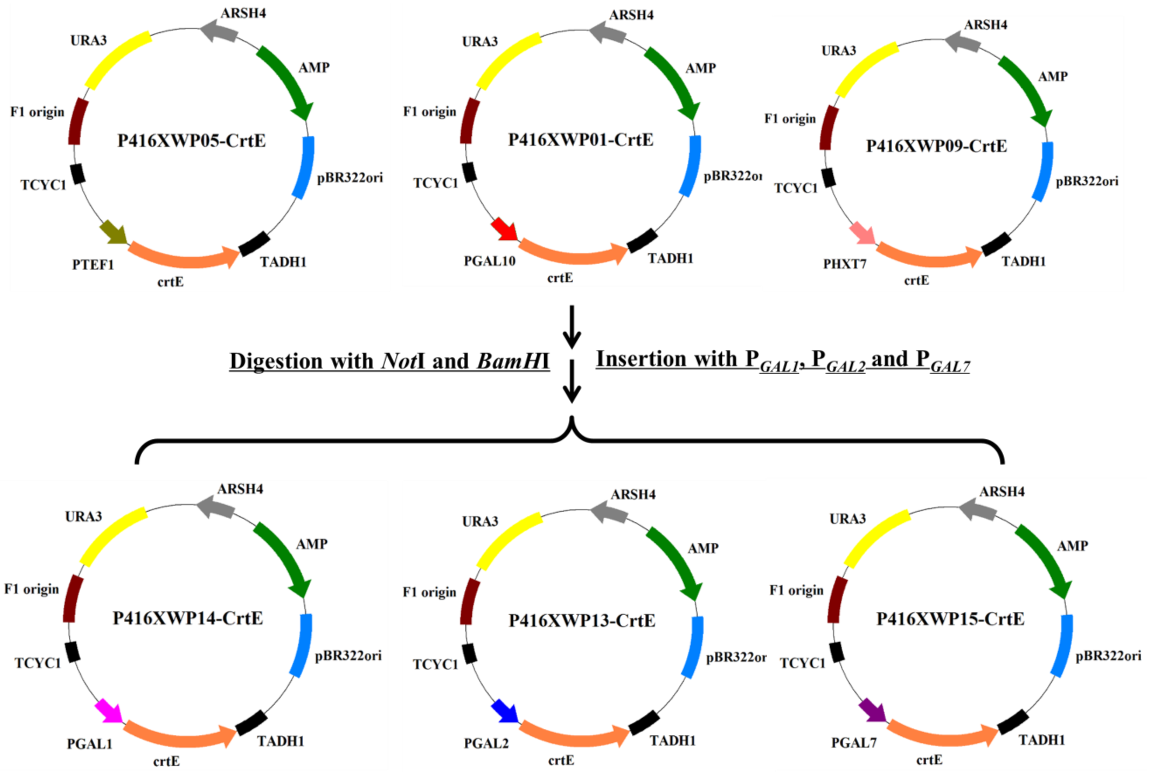

B

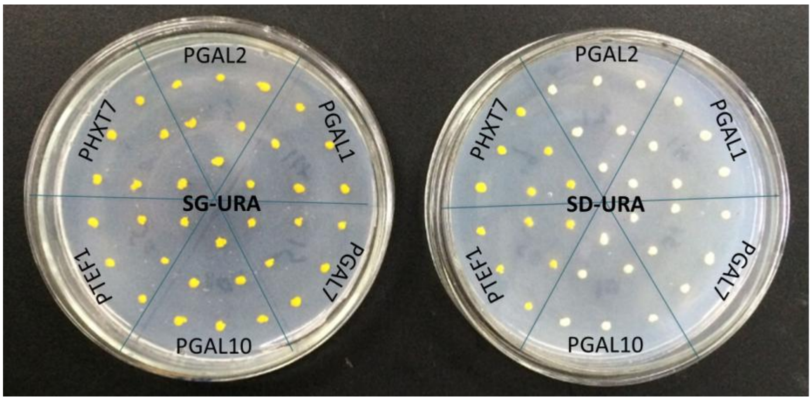

Supplementary Figure 2. Characterization of promoters.

A, Construction of P416XWP13/14/15-*CtrE* plasmids for promoter strength characterization. B, Strength comparison of promoters. Four GAL promoters (P_{GAL1}, P_{GAL10}, P_{GAL2} and P_{GAL7}) and two strong constitutive promoters (P_{TEF1} and P_{HXT7}) were tested using β-carotene as an indicator. The left plate shows YXWP41 derived strains harboring P416XWP-$P_{GAL1/GAL2/GAL7/GAL10/TEF1/HXT7}$-*CrtE* cultured on SG-URA (synthetic complete drop-out medium with 2% galactose and without uracil) plate, and the right plate shows the same strains cultured on SD-URA (synthetic complete drop-out medium with 2% D-glucose and without uracil) plate.

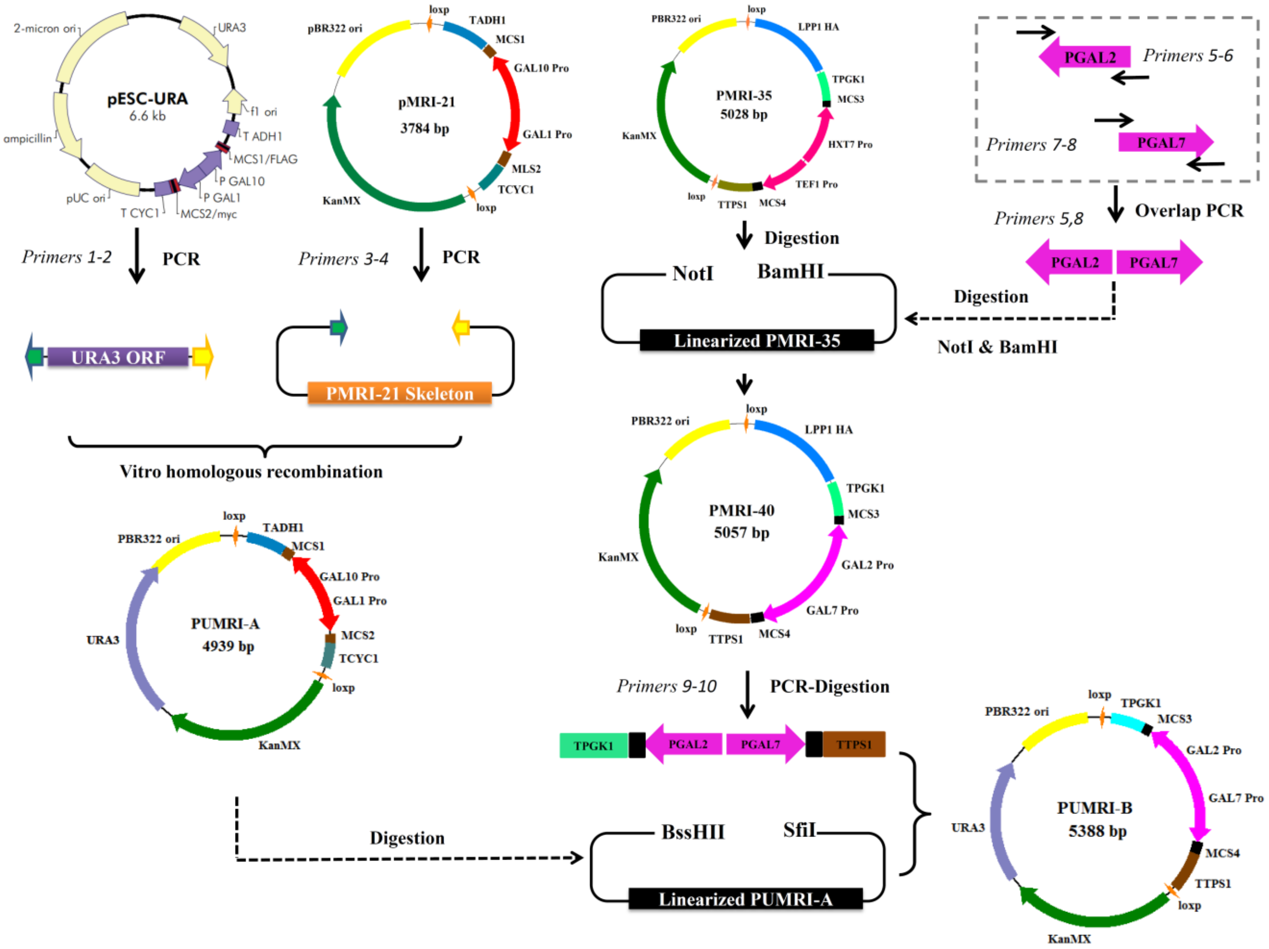

1

2 **Supplementary Figure 3. Construction process of the pUMRI toolbox**

3

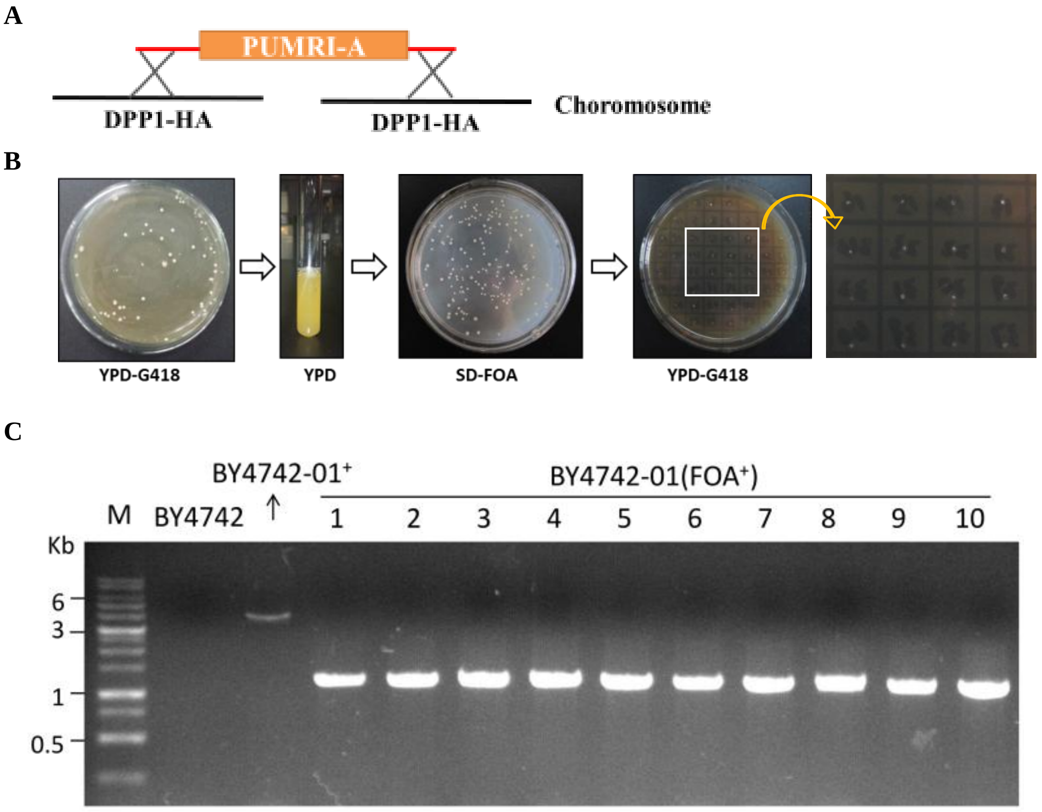

Supplementary Figure 4. Validation of the marker excision strategy.

A, Schematic representation of pUMRI-A integration in the *DPP1* site. B, Process and proof of marker excision based on double selection of FOA and G418 using BY4742-01^{+}. C, Colony PCR of BY4742-01^{+} and counterselection on FOA plates.

A

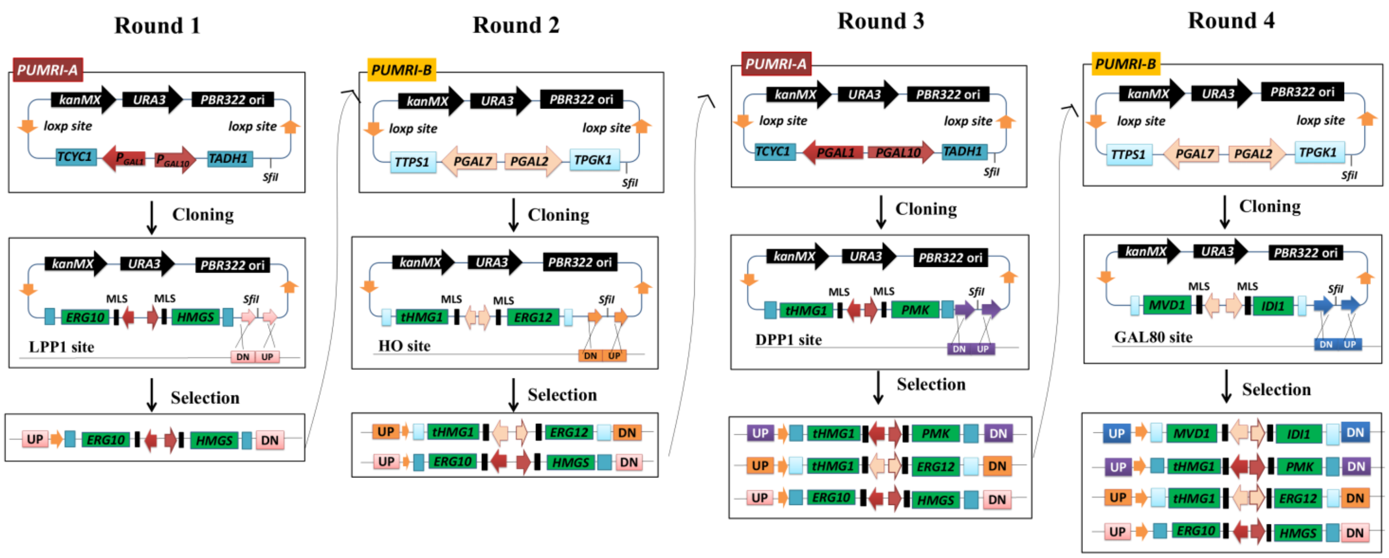

B

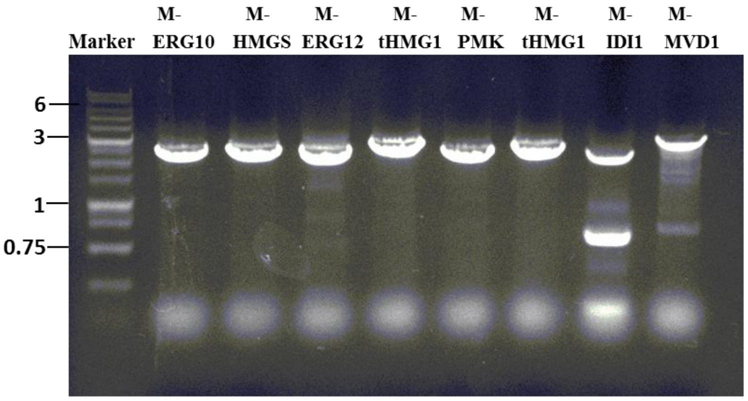

BY4742-M-04

Bands	ERG10	HMGS	ERG12	tHMG1-1	PMK	tHMG1-2	IDI1	MVD1
Primers	MLS26 -BamHI-F3	MLS26 -EcoRI-F3	MLS26 -EcoRI-F2	MLS26-BamHI-F2	MLS26 -EcoRI-F3	MLS26-BamHI-F3	MLS26-EcoRI-F2	MLS26-BamHI-F2
	LPP1UPF3	LPP1DR2	HO-R3	HO-F2	DPP1-DR2	DPP1-UPF2	GAL80UPF2	GAL80DR2
Expected size (kb)	2.5	2.4	2.5	2.7	2.4	2.5	2.5	3.3

C

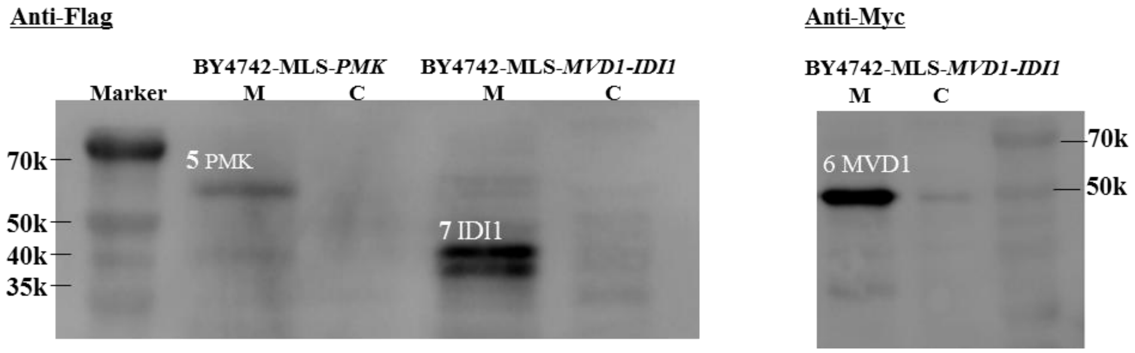

D

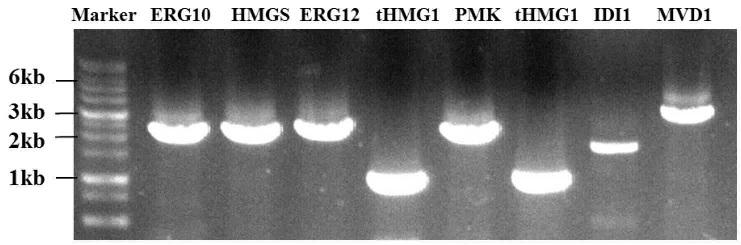

BY4742-C-04

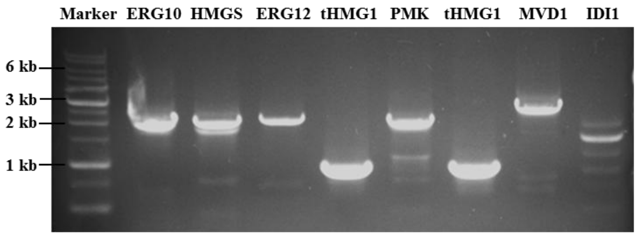

BY4741-C-04

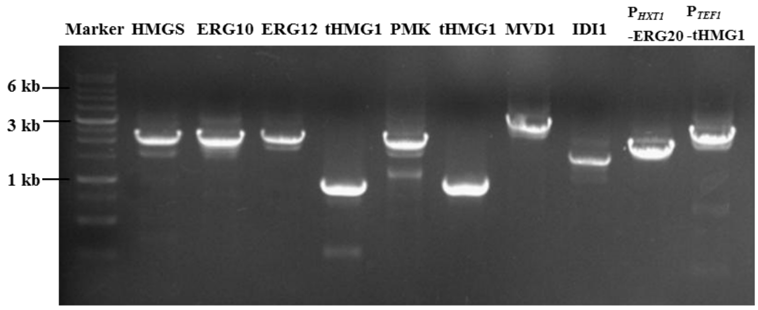

BY4742-C-05

Bands	ERG10	HMGS	ERG12	tHMG1-1	PMK	tHMG1-2	IDI1	MVD1	P_{HXT1}-ERG20	P_{TEF1}-tHMG1
Primers	ERG10-F1	HMGS-F1	ERG12-F1	qtHMG1-F1	PMK-F1	qtHMG1-F1	IDI1-F1-cx	MVD1-F1	ERG20R1	ERG20R1
	LPP1UPF3	LPP1DR2	HOR3	HO-F2	DPP1-DR2	DPP1-UPF2	GAL80UPF2	GAL80DR2	pHXT1F	tHMGR2
Expected size (kb)	2.5	2.4	2.5	1.1	2.4	1.1	1.8	3.3	2.1	2.7

Supplementary Figure 5. Construction and genotype analysis of BY4742-M/C-01/02/03/04.

A Reconstruction of a seven-gene MVA pathway in mitochondria via four rounds of integration. Details of the four rounds of reiterative recombination: The first round of recombination resulted in BY4742-M-01 (overexpression of *HMGS* and *ERG10* in BY4742), the second round of recombination resulted in BY4742-M-02 (overexpression of *tHMG1* and *ERG12* in BY4742-M-01), the third round of recombination resulted in BY4742-M-03 (overexpression of *PMK* and *tHMG1* in BY4742-M-02) and the fourth round of reiterative recombination resulted in BY4742-M-04 (overexpression of *MVD1* and *IDI1* in BY4742-M-03). B, Genotype analysis of BY4742-M-04 by PCR**.** The table below shows the theoretical sizes of the PCR fragments. C, Western blot analysis of the recombinant strains BY4742-MLS-*PMK* and BY4742-MLS-*MVD1-IDI1* for re-examination of PMK/IDI1/MVD1 expression. M: mitochondria, C: cytoplasm. D, Genotype analysis of BY4742-C-04, BY4741-C-04, and BY4742-C-05 by PCR. Table below shows the theoretical sizes of the PCR fragments.

A

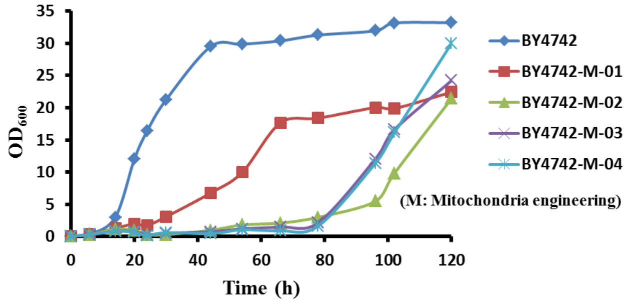

B

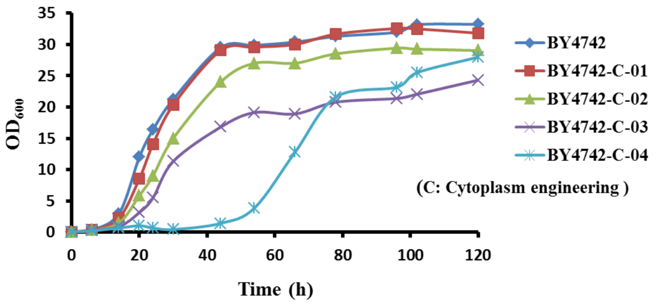

C

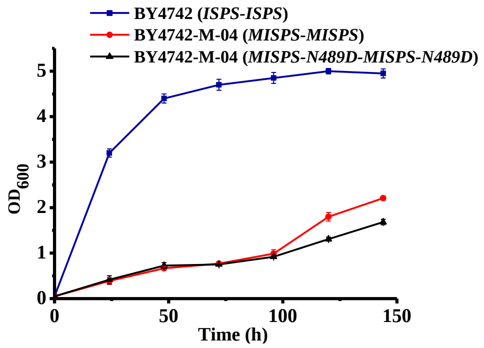

Supplementary Figure 6. Growth curves of the recombinant strains.

A, The cell growth curves of BY4742-M-01/02/03/04 (M: mitochondria engineering) cultured in 250 mL shake flasks with 50 mL YPG. B, The cell growth curves of BY4742-C-01/02/03/04 (C: cytoplasm engineering) cultured in 250 mL shake flasks with 50 mL YPG. C, The effect of *ISPS* overexpression on growth inhibition caused by pathway engineering. BY4742-M-04 harboring an inactive ISPS variant (N489D) and BY4742 (*ISPS-ISPS*) were used as the control strains for comparison. The recombinant strains were cultured in SG-URA for 144 h. The data in (A, B, C) are representative of three separate experiments. Bar represents mean ±s.d..

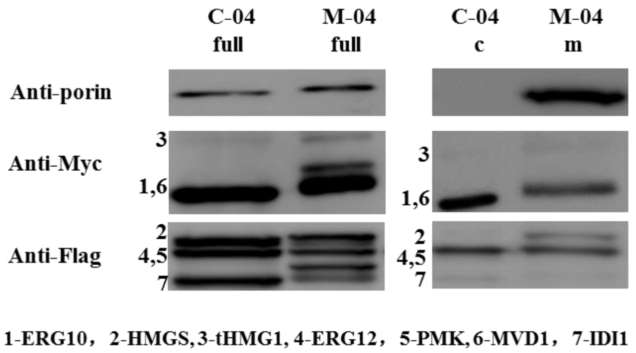

Supplementary Figure 7. Comparison of enzyme expression between BY4742-C-04 and BY4742-M-04 by western blot. Left channels are crude cell extracts of BY4742-C-04 and BY4742-M-04, right channels are the cytoplasm fraction of BY4742-C-04 and the mitochondria fraction of BY4742-M-04. Full-scanned images are shown in Supplementary Figure 9

A

ISPS		FS			CAS			LS			MS			SES	
position	AA	Pyrus communis	Mentha arvensis	Citrus junos	Matricaria chamomilla	Phyla dulcis	Artemisia annua	Mentha spicata	Cannabis sativa	Toona sinensis	Abies grandis	Alstroemeria peruviana	Picea abies	Ocimum basilicum	Abies grandis
**308**	R	R	R	R	R	R	R	R	R	R	R	R	R	R	R
**317**	W	C	W	W	W	W	W	W	W	W		W		W	W
**338**	F	I	V	V	I	I	I	N	Y	Y	C	I	C	I	I
**341**	V	V	C	I	A	A	A	I	L	I	V	A	V	V	C
**342**	T	L	G	T	T	S	T	T	T	T	T	T	T	A	T
**345**	D	D	D	D	D	D	D	D	D	D	D	D	D	D	D
**346**	D	D	D	D	D	D	D	D	D	D	D	D	D	D	D
**349**	D	D	D	D	D	D	D	D	D	D	D	D	D	D	D
**423**	E	E	E	E	E	E	E	E	E	E	E	E	E	E	E
**438**	Y	Y	Y	Y	Y	Y	Y	Y	Y	Y	Y	Y	Y	Y	Y
**447**	S	S	C	G	G	G	G	G	G	G	A	G	A	G	G
**485**	F	V	G	G	A	A	A	L	L	L	L	V	L	T	G
**486**	R	R	R	R	R	R	R	R	R	R	R	R	R	R	R
**489**	N	N	N	D	D	D	D	D	D	D	G	D	G	D	D
**490**	D	D	D	D	D	D	D	D	D	D	D	D	D	D	D
**497**	E	E	E	E	E	E	E	E	E	E	D	E	D	E	E
**505**	N	S	T	S	S	S	S	K	K	K	S	K	S	T	S

B

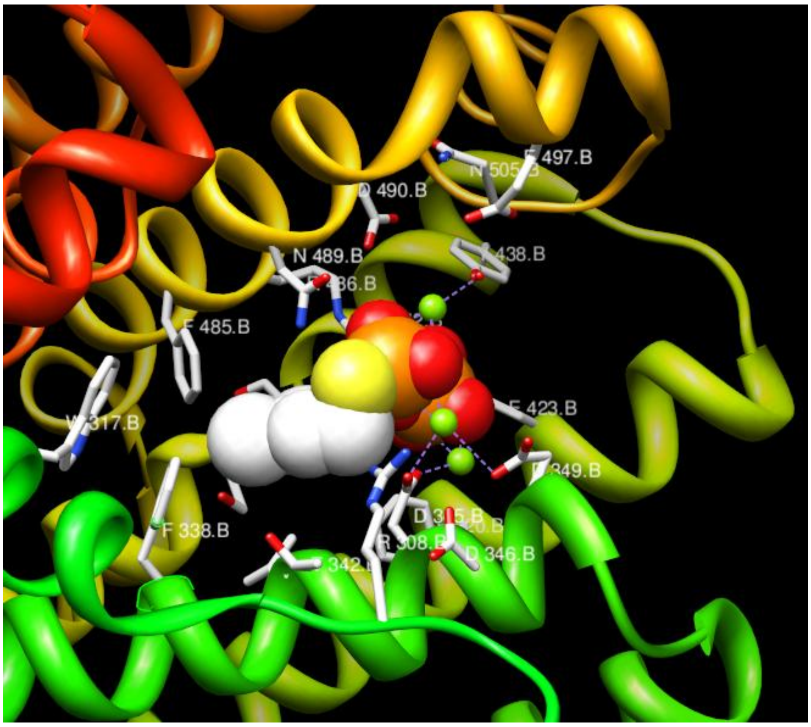

C

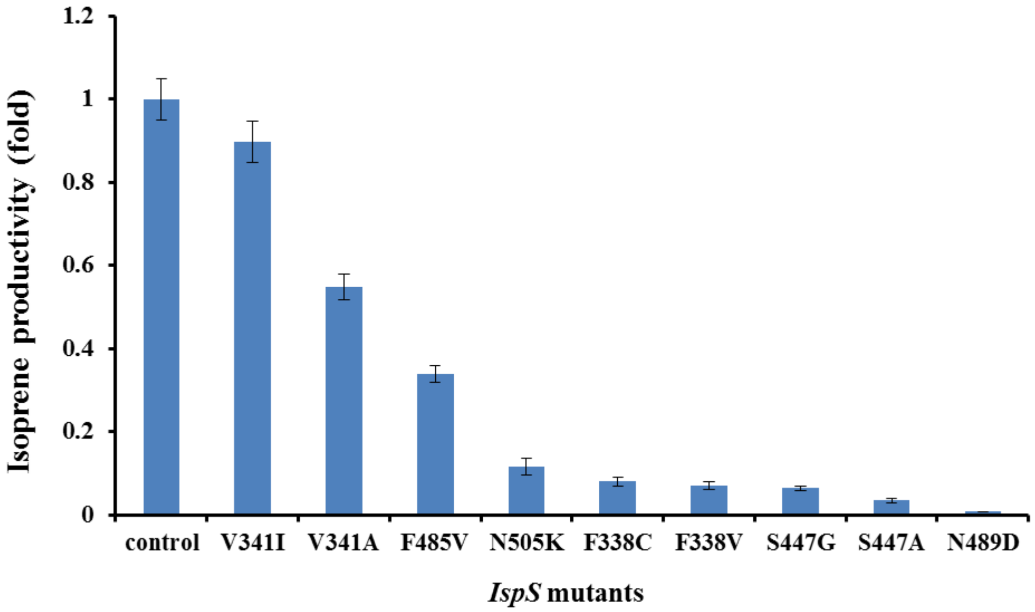

Supplementary Figure 8. Strategy and results of ISPS modification.

A, Sequence alignment between ISPS and other terpene synthases (FS: Farnesene synthase, CAS: Caryophyllene synthase, LS: Limonene synthase, MS: Myrcene synthase, SES: Selinene synthase). Highlighted in green are conserved amino acids. Highlighted in yellow (noncatalytic residues) and orange (catalytic residues) are residues in ISPS that are different from those in the other enzymes. B, Seventeen residues within 10 Å of the binding pocket of PcISPS are shown based on the X-ray crystal structure of the PcISPS-DMAPP analogue complex[1]. C, Isoprene productivity of ISPS mutants in YXM10. The production of isoprene in engineered strain YXM10 with wild-type ISPS was used as the reference for normalization. Error bars represent s.d. from three independent experiments.

Fig. 1D

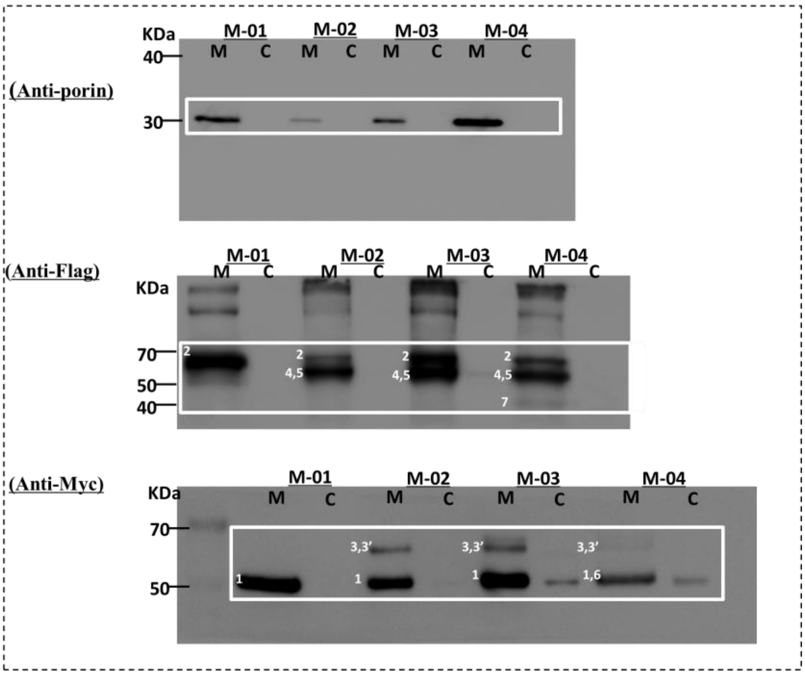

Suppl. Fig. 7

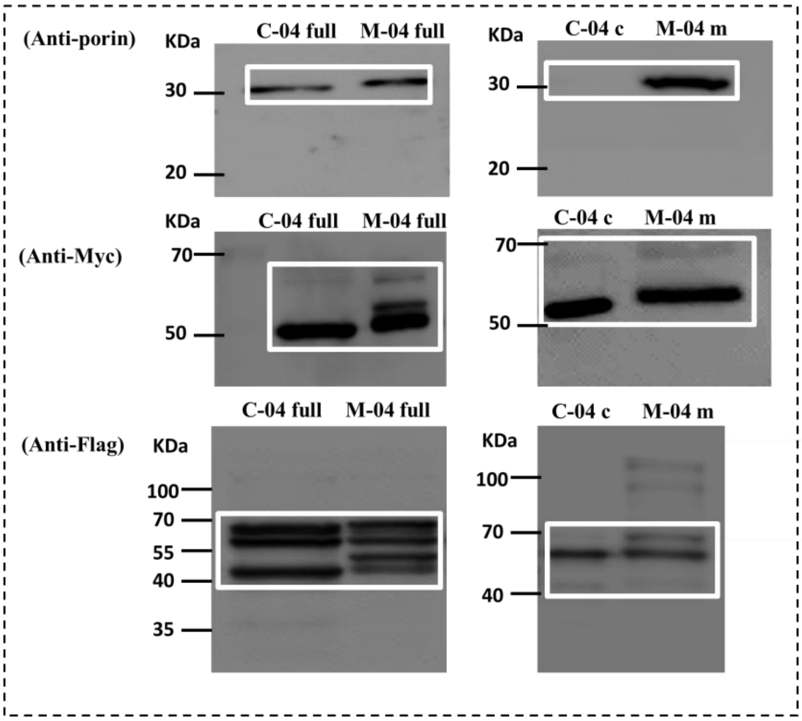

Supplementary Figure 9. Full-scanned images of western blots. White boxes show cropped regions.

Supplementary Tables

Supplementary Table 1. Marker (*loxp-KanMX-URA-PBR322-loxp*) loss frequency of transformants after FOA selection.

Strains	Volume of culture (μl)	No. of cells	No. of colonies on FOA plate	Frequency
C1	50	$4.9x10^{6}$	10	$2.0x10^{-6}$
C1	100	$9.8x10^{6}$	24	$2.4x10^{-6}$
C1	200	$19.6x10^{6}$	69	$3.5x10^{-6}$
C1	400	$39.2x10^{6}$	141	$3.6x10^{-6}$
C2	50	$3.9x10^{6}$	4	$1.0x10^{-6}$
C2	100	$7.8x10^{6}$	7	$9.0x10^{-7}$
C2	200	$15.6x10^{6}$	12	$7.7x10^{-7}$
C2	400	$31.2x10^{6}$	30	$9.6x10^{-7}$
C3	50	$4.45x10^{6}$	15	$3.4x10^{-6}$
C3	100	$8.9x10^{6}$	23	$2.6x10^{-6}$
C3	200	$17.8x10^{6}$	52	$2.9x10^{-6}$
C3	400	$35.6x10^{6}$	107	$3.0x10^{-6}$

Supplementary Table 2. Primers used in this work

Primers	Sequences
Primers used for amplifying GAL promoters	
PGAL7-F3	CGCGGATCCCTGTACCACCATGGAGACATCAAA
PGAL7-R3	ATAAGAATGCGGCCGCTTTTGAGGGAATATTCAACTGTTTT
PGAL1-F1-*BamH*I	CGCGGATCCGATGGACGCAAAGAAGTTTAATAAT
PGAL1-R1	ATAAGAATGCGGCCGCGTTTTTTCTCCTTGACGTTAAAGTATAG
PGAL2-F1-*BamH*I	GTGAGTCGTATTACGGATCCCTTCCGGAATGGCTTAAGTAGGTT
PGAL2-R1	ATAAGAATGCGGCCGCAGGGTTGAATTCTATGAAAGAATTATTTTTTTATTATGTTA
Primers for pUMRI toolbox construction (Numbering of primers is consistent with that in Supplementary Fig. 2A)	
1 URA(PRO-ORF-TER)-F2	AACGAACAGTGTGGCTGTGGTTCAGATTGTACTGAGAGTGCACCA
2 URA(PRO-ORF-TER)-R2	CGCCAGCAATCATTATAGAAATAACGTTTACAATTTCCTGATGCG
3 PMRI-21-F1	ATTTCTATAATGATTGCTGGCGTTTTTCCATAGGC
4 PMRI-21-R1	AACCACAGCCACACTGTTCGTTTTCGACACTGGAT
5 PGAL2-R1	ATAAGAATGCGGCCGCAGGGTTGAATTCTATGAAAGAATTATTTTTTTATTAT GTTA
6 PGAL2-F1	ATGGTGGTACAGCTTCCGGAATGGCTTAAGTAGGTT
7 PGAL7-F1	CCATTCCGGAAGCTGTACCACCATGGAGACATCAAA
8 PGAL7-R1	GTGAGTCGTATTACGGATCCTTTTGAGGGAATATTCAACTGTTTT
9 TPGK-GAL2/7-TTPS1-F1	TCTGGAGCTGGCCTTGTAGGCCTAACGAACGCAGAATTTTCGAGTTA
10 TPGK-GAL2/7-TTPS1-R1	ATTAAGGGTTGTCGAGCGCGCCGGT
Primers for pUMRI-A-DPP1 construction and genotype identification of BY4742-01+ and BY4742-01	
*DPP*1-UPF1	CCATCAGGCCTTTATGGCCGCATTATGTCCGATAAACACAG
*DPP*1-UPR1	GAACAAAAGCTGGAGCTGGCCTTGTCAACCGATCGACAAATTATTTC
*DPP*1-DF1	GGCGTAATAGCGAAGAGGCCTACAGAAGGCTTGCCATTGGACAC
*DPP*1-DR1	CATAATGCGGCcataaagGCCTGATGGGTGACTGCTTCCTC
*DPP*1-UPF2	AAAGGGACAACACGGCTTATA
*GAL*1F	TCTGGGGTAATTAATCAGCG
Primers used for strain construction	
MLS26-EcoRI-F3	GTAAGAATTTTTGAAAATTCGAATTATGCTTTCACTACGTCAATCTATAAGATTT TT

MLS26-EcoRI-R3	GCCGCCCTTTAGTGAGGGTTGAATTCCTGAAGCAGATATCTAGAGCTACACAAAG
MLS26-BamHI-F3	CGTCAAGGAGAAAAAACCCCGGATCATGCTTTCACTACGTCAATCTATAAGATTTTT
MLS26-BamHI-R2	CCTATAGTGAGTCGTATTACGGATCCCTGAAGCAGATATCTAGAGCTACACAAAG
MLS26-EcoRI-F2	AAAAAAATAATTCTTTCATAGAATTATGCTTTCACTACGTCAATCTATAAGATTTTT
MLS26-EcoRI-R2	ACTAGTGCGGCCGCAGGGTTGAATTCCTGAAGCAGATATCTAGAGCTACACAAAG
MLS26-BamHI-F2	AGTTGAATATTCCCTCAAAAGGATCATGCTTTCACTACGTCAATCTATAAGATTTTT
ERG10-F1	AGTACGCGGATCCATGTCTCAGAACGTTTACATTGTATCG
ERG10-R1	TGTTCCATGTCGACTATCTTTTCAATGACAATAGAGGAAGC
HMGS-F1	AGTGCCGGAATTCATGAAACTCTCAACTAAACTTTGTTGGT
HMGS-R1	ATAGTTTAGCGGCCGCTTTTTTAACATCGTAAGATCTTCTAAAT
tHMG1-F1	CTATCGCGGATCCAAAAATGGACCAATTGGTGAAAACTGA
tHMG1-F2	CTATCGCGGATCCGACCAATTGGTGAAAACTGA
tHMG1-R1	ATCGTCCCCCCGGGAAGGATTTAATGCAGGTGACGGA
tHMG1-R2	TGTTCCATGTCGACGGATTTAATGCAGGTGACGGA
ERG12-F1	AAAAAATAATTCTTTCATAGAATTCATGTCATTACCGTTCTTAACTTCTG
ERG12-F2	TGTGTAGCTCTAGATATCTGCTTCAGGAATTCATGTCATTACCGTTCTTAACTTCTG
ERG12-R1	CATCCTTGTAATCCATCGATACTAGTGATGAAGTCCATGGTAAATTCGTGT
PMK-F1	AGTGCCGGAATTCATGTCAGAGTTGAGAGCCTTCAGTGC
PMK-R1	ATAGTTTAGCGGCCGCTTTATCAAGATAAGTTTCCGGATC
MVD1-F1	CTATCGCGGATCCATGACCGTTTACACAGCATCCGTTA
MVD1-R1	ATCGTCCCCCCGGGAATTCCTTTGGTAGACCAGTCTTTGC
IDI1-F2	CATAGAATTCAACCCTGCGGCCGCATGACTGCCGACAACAATAGTATGC
IDI1-F3	CAACCCTGCGGCCGCCATGACTGCCGACAACAATAGTATGC
IDI1-R1	CATCCTTGTAATCCATCGATACTAGTGATAGCATTCTATGAATTTGCCTGTC

LPP1-UpF1	CCGATGGCCTTTATGGCCAATCATGGTTTCATGGTCACT
LPP1-UpR1	GAACAAAAGCTGGAGCTGGCCTTGTAAATTCTTACGCGTGAACCAAAA
LPP1-DF1	GGCGTAATAGCGAAGAGGCCTACATGGAGCTGTTCTAGCATTTTT
LPP1-DR1	GATTGGCCATAAAGGCCATCGGTATTTTGGCTTCGGT
DPP1-UPF1	CCATCAGGCCTTTATGGCCGCATTATGTCCGATAAACACAG
DPP1-UPR1	GAACAAAAGCTGGAGCTGGCCTTGTCAACCGATCGACAAATTATTTC
DPP1-DF1	GGCGTAATAGCGAAGAGGCCTACAGAAGGCTTGCCATTGGACAC
DPP1-DR1	CATAATGCGGCcataaagGCCTGATGGGTGACTGCTTCCTC
HOleftF1	GATGCTGTCCGCGGGCCTCATGGGCCGAATCGCGTAAAAAG
HOleftR1	GAACAAAAGCTGGAGCTGGCCTTGTATCGAGATCACTTTTCGTG
HOrightF2	ACTCGAAAATTCTGCGTTCGTTAGGCCTACAAGACGACCAGGTCAGCTAGGGAG
HOrightR1	CTTTTTACGCGATTCGGCCCATGAGGCCCGCGGACAGCATC
GAL80-UP-F1	GAAGGCGATGCCGGCCATAAAGGCCCGAACGACCTCAAAATG
GAL80-UP-R1	CTCGAAAATTCTGCGTTCGTTAGGCCTACAATTCCAAGAGAGGCATAACAACTTCAT
GAL80-DN-F1	ACTAAAGGGAACAAAAGCTGGAGCTGGCCTTGTAACGTGGGGTTCAAACCATCAT
GAL80-DN-R1	GAGGTCGTTCGGGCCTTTATGGCCGGCATCGCCTTCAAGTTT
HIS3-F1	CGTTTTAAGAGCTTGGTGAGC
HIS3-R1	CGCCTCGTTCAGAATGACA
Primers for genotype analysis of recombinant strains	
HMGS-F1	AGTGCCGGAATTCATGAAACTCTCAACTAAACTTTGTTGGT
LPP1DR2	CTTATACGCCTCAAGACGACA
LPP1UPF3	TGTCAATGAGTTTTCGCAGA
ERG10-F1	AGTACGCGGATCCATGTCTCAGAACGTTTACATTGTATCG
ERG12-F1	AAAAAATAATTCTTTCATAGAATTCATGTCATTACCGTTCTTAACTTCTG
HOR3	GGCGTATTTCTACTCCAGCA
MLS26-BamHI-F2	AGTTGAATATTCCCTCAAAAGGATCATGCTTTCACTACGTCAATCTATAAGATTTTT
HO-F2	CGTGCCTGCGATGAGATAC

PMK-F1	AGTGCCGGAATTCATGTCAGAGTTGAGAGCCTTCAGTGC
DPP1-DR2	CAAGCAGGCCCTTGCACGTC
DPP1-UP-F2	AAAGGGACAACACGGCTTATA
MLS26-BamHI-F3	CGTCAAGGAGAAAAAACCCCGGATCATGCTTTCACTACGTCAATCTATAAGATTTTT
GAL80UPF2	GGATAAAGACGGGTCGGATAC
MLS26-EcoRI-F2	AAAAAAATAATTCTTTCATAGAATTATGCTTTCACTACGTCAATCTATAAGATTTTT
GAL80DR2	AATAAAGCCATTCATCGTGTTG
MLS26-EcoRI-F3	GTAAGAATTTTTGAAAATTCGAATTATGCTTTCACTACGTCAATCTATAAGATTTTT
qtHMG1-F1	CCTAACAATTTGGACGCCAC
tHMG1R2	GCGCAGATGATAAACTTTTGAC
Erg20R1	GGAAGTCGACCTATTTGCTTCTCTTGTAAACTTTG
IDI1-F1-cx	TACGCCTTGGTTTAAGATTATTTGC
MVD1-F1	CTATCGCGGATCCATGACCGTTTACACAGCATCCGTTA
pHXT1F	GCGGGATCCTGCAGGTCTCATCTGGAATATAATTCC

Supplementary Table 3. List of all strains and plasmids

Strains	Genotype	Plasmids	Source
BY4742	*MATα, his3Δ1, leu2Δ0, lys2Δ0, ura3Δ0*	None	Ref.[2]
BY4742-01+	BY4742, Δ*DPP1*::T_{ADH1}-P_{GAL10}-P_{GAL1}-T_{CYC1}-(*Kan*MX-*URA3-PBR322ori*)	None	This study
BY4742-01	BY4742, ΔDPP1::T_{ADH1}-P_{GAL10}-P_{GAL1}-T_{CYC1}	None	This study
YXWP41	BY4741, HO::T_{ADH1}-crtYB-P_{GAL10}-P_{GAL1}-crtI-T_{CYC1}; YPRCtau3::T_{ADH1}-crtYB-P_{GAL10}-P_{GAL1}-crtI-T_{CYC1}	None	Ref.[3]
YXWP41-01-*CrtE*	YXMP41	P416XWP01-CrtE	Ref.[3]
YXWP41-05-*CrtE*	YXMP41	P416XWP05-CrtE	Ref.[3]
YXWP41-09-*CrtE*	YXMP41	P416XWP09-*CrtE*	Ref.[3]
YXWP41-13-*CrtE*	YXMP41	P416XWP13-*CrtE*	This study
YXWP41-14-*CrtE*	YXMP41	P416XWP14-*CrtE*	This study
YXWP41-15-*CrtE*	YXMP41	P416XWP15-*CrtE*	This study
BY4742-C-01	BY4742, Δ*LPP1:* T_{CYC1}-*ERG10*-P_{GAL1}-P_{GAL10}-*HMGS*-T_{ADH1}	None	This study
BY4742-C-02	BY4742-C-01, Δ*HO:* T_{TPS1}-*tHMG1*-P_{GAL7}-P_{GAL2}-*ERG12*-T_{PGK1}	None	This study
BY4742-C-03	BY4742-C-02, Δ*DPP1:* T_{CYC1}-*tHMG1*-P_{GAL1}-P_{GAL10}-*PMK*-T_{ADH1}	None	This study
BY4742-C-04	BY4742-C-03, Δ*GAL80:* T_{TPS1}-*MVD1*-P_{GAL7}-P_{GAL2}-*IDI1*-T_{PGK1}	None	This study
BY4741-C-04	BY4741, Δ*LPP1:* T_{CYC1}-*ERG10*-P_{GAL1}-P_{GAL10}-*HMGS*-T_{ADH1}; Δ*HO:* T_{TPS1}-*tHMG1*-P_{GAL7}-P_{GAL2}-*ERG12*-T_{PGK1}; Δ*DPP1:* T_{CYC1}-*tHMG1*-P_{GAL1}-P_{GAL10}-*PMK*-T_{ADH1}; Δ*GAL80:* T_{TPS1}-*MVD1*-P_{GAL7}-P_{GAL2}-*IDI1*-T_{PGK1}	None	This study
BY4742-M-01	BY4742, Δ*LPP1:* T_{CYC1}- *ERG10-MLS*-P_{GAL1}-P_{GAL10}-*MLS-HMGS*-T_{ADH1}	None	This study
BY4742-M-02	BY4742-M-01, Δ*HO:* T_{TPS1}- *tHMG1-MLS*-P_{GAL7}-P_{GAL2}-*MLS-ERG12*-T_{PGK1}	None	This study
BY4742-M-03	BY4742-M-02, Δ*DPP1:* T_{CYC1}- *tHMG1-MLS*-P_{GAL1}-P_{GAL10}-*MLS-PMK*-T_{ADH1}	None	This study
BY4742-M-04	BY4742-M-03, Δ*GAL80:* T_{TPS1}- *MVD1-MLS*-P_{GAL7}-P_{GAL2}-*MLS-IDI1*-T_{PGK1}	None	This study
BY4742-MC-01	BY4742-M-04, ΔP_{ERG20}-*ERG20*::P_{HXT1}-*ERG20*-P_{TEF1}-*tHMG1*	None	This study
BY4742-C-05	BY4742-C-04, ΔP_{ERG20}-*ERG20*::P_{HXT1}-*ERG20*-P_{TEF1}-*tHMG1*	None	This study
YXM10	BY4741, (*HMG1*)::*tHMG1*, △P_{ERG20}::P_{HXT1}, Ty4::*ERG10-ACS2* △*GAL80::LEU2*	None	Ref.[4]
YXMH-01	Diploid BY4742-ΔGAL80::HIS xYXM10	None	This study
YXMH-02	Diploid BY4742-M-04-HIS x BY4741-ΔGAL80::LEU	None	This study
YXMH-03	Diploid BY4742-M-04-HIS x YXM10	None	This study
YXMH-04	Diploid BY4742-M-04-HIS x BY4741-C-04-LEU	None	This study
BY4742-M-01 (*MISPS-MIPS*)	BY4742-M-01	pESC-URA-*MISPS-MISPS*	This study
BY4742-M-02 (*MISPS-MISPS*)	BY4742-M-02	pESC-URA-*MISPS-MISPS*	This study

BY4742-M-03 (*MISPS-MISPS*)	BY4742-M-03	pESC-URA-*MISPS-MISPS*	This study
BY4742-M-04 (*MISPS-MISPS*)	BY4742-M-04	pESC-URA-*MISPS-MISPS*	This study
BY4742-C-05 (*ISPS-ISPS*)	BY4742-C-05	pESC-URA-*ISPS-ISPS*	This study
BY4742-MC-01 (*ISPS-MISPS*)	BY4742-MC-01	pESC-URA-*ISPS-MISPS*	This study
YXMH-01 (*ISPS-ISPS*)	YXMH-01	pESC-URA-*ISPS-ISPS*	This study
YXMH-02 (*MISPS-MISPS*)	YXMH-02	pESC-URA-*MISPS-MISPS*	This study
YXMH-03 (*ISPS-MISPS*)	YXMH-03	pESC-URA-*ISPS-MISPS*	This study
YXMH-04 (*ISPS-MISPS*)	YXMH-04	pESC-URA-*ISPS-MISPS*	This study

Supplementary Methods

Marker recyclable integration of the pUMRI toolbox

1. Gene cloning: The target genes were inserted into the MCS of the pUMRI plasmids. Homologous arms were selected based on the reported chromosome fragments[5] that are dispensable for cell growth, designed into two parts (up-arm and down-arm) and then inserted into the *Sfi*I site of the toolbox. *E. coli* DH5α was selected as the host for cloning, and transformation was performed using the heat shock method.

2. Transformant selection: The recombinant plasmids were linearized by *Sfi*I digestion and then transformed into yeast strains using electroporation. The transformants were spread on YPD-G418 or SD-URA plates. The positive recombinant strains were identified by double selection based on the G418 and URA markers in pUMRI.

3. Marker recycling: Colonies of positive strains were picked from the YPD-G418 or SD-URA plates, inoculated into YPD medium and cultivated at 30 ^{o}C for 24 h. The cells were collected, washed twice with sterile water and spread on SD-FOA plates. The strains that could survive on SD-FOA plates were probably the recombinants with marker excision. These results were then verified by inoculating the strains on G418 plates. The strain with marker excision was used as the host for the next round of integration.

Validation of the marker excision strategy.

To determine the efficiency of the marker excision strategy, *DPP1* deletion was performed. First, pUMRI-A was modified by insertion of *DPP*1 (Supplementary Fig. 4A), linearized by *Sfi*I, transformed into BY4742 and selected on geneticin (G418) plates, generating the strain BY4742-01$^+$. Correct fragment integration was verified by colony PCR, giving a 4.7 kb-band (Supplementary Fig. 4C). To obtain the *KanMX-URA* excised transformant, BY4742-01$^+$ was cultured at 30 $^{\circ}$C for 24 h and then directly spread on FOA plates. A total of 52 colonies were picked out and replica-inoculated onto G418 plate. No colony survived (Supplementary Fig. 4B). To further confirm the successful excision of the marker, 10 colonies were randomly picked from FOA plate and examined by colony PCR. As shown in Supplementary Fig. 4C (BY4742-01, 1-10), all colony-PCR bands showed the expected size of 1.2 kb, indicating that *KanMX-URA* was indeed removed.

To evaluate the loss frequency of the marker, three independent single colonies (C1, C2, C3) were randomly picked from the G418 plate (BY4742-01$^+$) and subjected to FOA selection. Cells were cultured for 24 h and then spread on FOA plates. In addition, diluted cell suspensions were spread on YPD (1% Yeast extract, 2% Peptone and 2% D-glucose) plates to count the total colony number. The loss frequency was calculated by dividing the colony number on the FOA plates by the total colony number. The recombination frequency between *loxp* repeats was determined to be 2.2×10^{-6} (Table S1), which was rather low compared to that of the *Cre/loxp* system (about 70%).

ISPS modification

Based on sequence alignment with similar terpene synthases (farnesene synthase, caryophyllene synthase, limonene synthase, myrcene synthase and selinene synthase) (Supplementary Fig. 8A), 6 residues located in the binding pocket were identified as

potentially important, among which 3 noncatalytic residues (V341, F485, F338) were modified to decrease the steric hindrance and 3 catalytic residues (N505, S447, N489) were engineered to change the binding forces between the enzyme and the substrate (Supplementary Fig. 8B). The results are shown in Supplementary Fig. 8C. According to the X-ray crystal structure of PcISPS (isoprene synthase from gray poplar hybrid *Populus×canescens*)[6], the unreactive substrate analogue DMASPP (dimethylallyl-S-thiolodiphosphate) diphosphate group forms hydrogen bonds with R486 and N489, in addition to its interactions with metal ions (R486 is conserved in monoterpene cyclases BPPS and LMNS, whereas N489 is an aspartic acid in these cyclases). The almost complete activity loss of ISPS-N489D suggested that N489 might be a key residue in the catalysis of ISPS. Noncatalytic residues (V341, F485, F338) form van der Waals interaction with DMASPP, resulting in a shallower hydrophobic pocket in comparison to those of other terpene cyclases. Generally, decrease of steric hindrance in the binding pocket can improve enzyme activity, while the activity of V341I/V341A/F485V/N505K/F338C/F338G strains were decreased, indicating that the specific spatial structure of the shallower hydrophobic pocket might benefit the stabilization of transition state configuration rather than hinder the binding. As part of the future work, rational design methods like dynamic simulation can be employed for ISPS engineering.

Reconstruction of the MVA pathway

The 26 AA N-terminal mitochondrial localization signal (MLS) (sequence: ATGCTTTCACTACGTCAATCTATAAGATTTTTCAAGCCAGCCACAAGAACTTTGTGTAGCTCTAGATATCTGCTTCAG) from subunit IV of yeast cytochrome oxidase (CoxIV) (GenBank No. 001181052) was amplified from the genome of BY4742 with MLS26-*EcoR*I-F3 and MLS26-*EcoR*I-R3, MLS26-*BamH*I-F3 and MLS26-*BamH*I-R2, and cloned into pUMRI-A, generating pUMRI-A-MLS. The same sequence was amplified with MLS26-*EcoR*I-F2 and MLS26-*EcoR*I-R2, MLS26-*BamH*I-F2 and MLS26-*BamH*I-R2, and cloned into pUMRI-B, generating pUMRI-B-MLS. *HMGS* and *ERG10* were amplified with primer pairs of *HMGS-F1&HMGS-R1 and ERG10-F1&ERG10-R1*, digested with *EcoR*I/*Not*I and *BamH*I/*Sal*I, respectively, and then inserted into the corresponding sites of pUMRI-A-MLS, generating pUMRI-A-MLS-*HMGS-ERG10*. Up homologous arm of *LPP1* was amplified with the primer pair *LPP1-UpF1*&*LPP1-UpR1*, generating *LPP1-UP*. Down homologous arm of *LPP1* was amplified with the primer pair *LPP1-DF1*&*LPP1-DR1*, generating *LPP1-Down*. The *LPP1* homologous arms were obtained by overlap extension PCR using *LPP1-UpR1* and *LPP1-DF1* as the primers and *LPP1-UP* and *LPP1-Down* as the templates. The fused segment was integrated into the *Sfi*I site of pUMRI-A-MLS-*HMGS-ERG10* by *in vitro* recombination, generating pUMRI-A-MLS-Δ*LPP1-HMGS-ERG10*. Similarly, recombinant plasmids containing the other six genes were constructed and integrated into BY4742 to generate BY4742-M-01/02/03/04. The same process was performed for BY4742-C-01/02/03/04 construction using pUMRI plasmids, with the exception of MLS introduction. All the primers are listed in Table S2.

Supplementary References

1 **Koksal, M., Zimmer, I., Schnitzler, J. P. & Christianson, D. W. Structure of Isoprene Synthase Illuminates the Chemical Mechanism of Teragram Atmospheric Carbon Emission. *Journal of Molecular Biology* 402, 363-373 (2010).**

2 **Brachmann, C. B. *et al.* Designer deletion strains derived from Saccharomyces cerevisiae S288C: a useful set of strains and plasmids for PCR-mediated gene disruption and other applications. *Yeast* 14, 115-132 (1998).**

3 **Xie, W., Ye, L., Lv, X., Xu, H. & Yu, H. Sequential control of biosynthetic pathways for balanced utilization of metabolic intermediates in *Saccharomyces cerevisiae*. *Metab Eng* 28C, 8-18 (2014).**

4 **Lv, X. *et al.* Enhanced isoprene biosynthesis in Saccharomyces cerevisiae by engineering of the native acetyl-CoA and mevalonic acid pathways with a push-pull-restrain strategy. *J Biotechnol* 186C, 128-136 (2014).**

5 **Winzeler, E. A. *et al.* Functional characterization of the *S-cerevisiae* genome by gene deletion and parallel analysis. *Science* 285, 901-906 (1999).**

6 **Koksal, M., Zimmer, I., Schnitzler, J. P. & Christianson, D. W. Structure of isoprene synthase illuminates the chemical mechanism of teragram atmospheric carbon emission. *J Mol Biol* 402, 363-373 (2010).**
